# Ready Access to Molecular Rotors Based on Boron Dipyrromethene Dyes-Coumarin Dyads Featuring Broadband Absorption

**DOI:** 10.3390/molecules25040781

**Published:** 2020-02-12

**Authors:** Ernesto Enríquez-Palacios, Teresa Arbeloa, Jorge Bañuelos, Claudia I. Bautista-Hernández, José G. Becerra-González, Iñigo López-Arbeloa, Eduardo Peña-Cabrera

**Affiliations:** 1 Departamento de Química, Universidad de Guanajuato. Noria Alta S/N. Guanajuato, Gto. 36050, Mexico; e.enriquezpalacios@ugto.mx (E.E.-P.); cla_isb_6@hotmail.com (C.I.B.-H.); jg.becerragonzalez@ugto.mx (J.G.B.-G.); 2 Departamento de Química Física. Universidad del País Vasco-EHU, Apartado 644, 48080 Bilbao, Spain; teresa.arbeloa@ehu.es (T.A.); inigo.lopezarbeloa@ehu.es (I.L.-A.)

**Keywords:** molecular rotors, BODIPY, viscosity sensors, dye chemistry, energy-electron transfer

## Abstract

Herein we report on a straightforward access method for boron dipyrromethene dyes (BODIPYs)-coumarin hybrids linked through their respective 8- and 6- positions, with wide functionalization of the coumarin fragment, using salicylaldehyde as a versatile building block. The computationally-assisted photophysical study unveils broadband absorption upon proper functionalization of the coumarin, as well as the key role of the conformational freedom of the coumarin appended at the *meso* position of the BODIPY. Such free motion almost suppresses the fluorescence signal, but enables us to apply these dyads as molecular rotors to monitor the surrounding microviscosity.

## 1. Introduction

**Figure 1:**
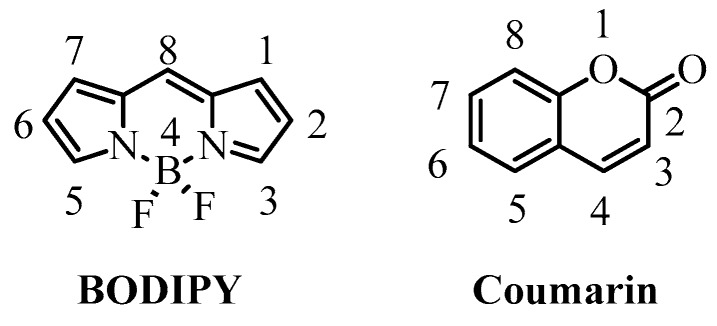
Structure of boron dipyrromethene dyes (BODIPYs) and coumarins.

The design of molecules featuring two or more chromophoric units is of great interest [1,2,3,4]. One can envisage new applications of such systems by taking advantage of the interactions that may develop between the chromophoric units, i.e., energy transfer [5,6], or electron transfer pathways [7], enabling the design of light harvesters with broadband absorption and high *pseudo*-Stokes shifts [8,9,10,11], or photosensitizers for photovoltaic devices [12,13] mimicking the natural photosynthesis [14,15]. It is crucial to the design of multichromophoric systems to have flexible functionalization methods at one’s disposal that are able to tailor the targeted analogues. In this regard, both boron dipyrromethene dyes (BODIPYs) [16] and coumarins [17] are in themselves two of the more widely used fluorophores (Figure 1), and hence have optimal candidates to design multichromophore ongoing energy transfer processes. Indeed, the former luminophores stand out due to their stability, chemical versatility and tunable photophysical signatures [18,19], whereas the latter fluorophores can be also deeply functionalized and display spectral bands at higher energies than the BODIPY core. Thus, both chromophores are complementary from a spectral point of view, and hence are suitable building blocks to be combined in a single molecular structure towards the promotion of intramolecular energy transfer hops.

Despite the fact that coumarin-BODIPY hybrids are known, and some examples of these dyads can be found in the literature applied as chemosensors [20,21], energy transfer cassettes [22,23] or fluorescent probes for bioimaging [24,25], the photonic performance, and hence the practical applicability of these multifunctional molecular assemblies, can be still improved. Indeed, one of the main drawbacks of these multichromophores is the low absorbance of the coumarin core [26], which falls in the ultraviolet (UV) region and it is usually masked (or at least overlapped) under the more energetic transitions of the BODIPY unit. This feature hampers the application of these dyads, for instance as broadband energy transfer cassettes since, while the energy transfer from the coumarin to the BODIPY is effective, the light harvesting is not greatly improved by the presence of coumarin.

From the synthetic point of view, the utilization of salicylaldehyde as a versatile building block is well-documented [27]. Herein, we show that such an attractive starting material can be rendered fluorescent by attaching it to a BODIPY fragment, thereby opening up new possibilities for the synthesis of more complex products. In this first example, we used the BODIPY-containing salicylaldehyde to prepare BODIPY-coumarin hybrids. Thanks to this methodology, we have been able to decorate the chromene π-system of the coumarin with a battery of aromatic moieties (from functionalized aryl groups with electron donors, i.e., methoxy, or acceptors, i.e., cyano, groups, to pyridine, naphthalene, modified stilbenes, triphenylamine, or benzothiophene). The computationally-aided spectroscopic analysis of this set of dyads allows the selection of the best structural modification at the coumarin subunit to enhance the light harvesting ability along the UV-yellow spectral region towards applying these dyads as energy transfer cassettes.

Furthermore, in view of the conformational flexibility of these dyads around the linking bond between the 8-position of the BODIPY and the coumarin, we anticipated that they could behave as molecular rotors [28,29,30]. Thus, we have conducted additional measurements at different temperatures and controlled viscosities (increasing the amount of ethylene glycol in the medium) to test the viability of these hybrids as fluorescent sensors to monitor the viscosity of the surrounding environment.

## 2. Results and Discussion

### 2.1. Synthesis

**Scheme 1:**
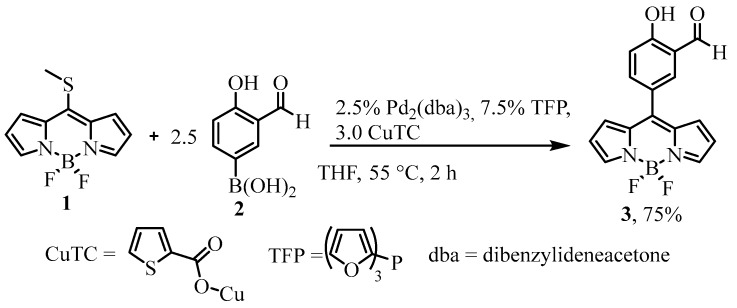
LSCC reaction conditions.

Salicyladehyde was attached to BODIPY via the Liebeskind-Srogl cross-coupling (LSCC) reaction [31] between commercially available 8-methylthioBODIPY **1** and boronic acid **2** (Scheme 1).

**Scheme 2:**
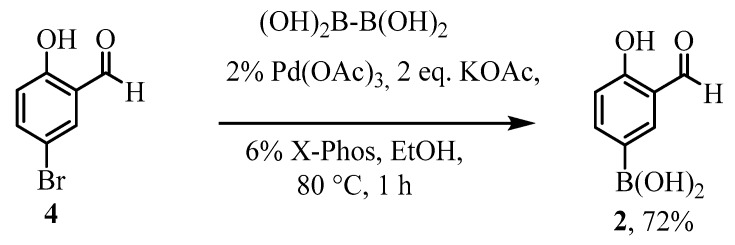
Synthetic access to the boronic acid precursor.

Key boronic acid **2** was prepared by treating commercially available bromide **4** with diboronic acid in the presence of Pd (Scheme 2) [32].

**Scheme 3:**
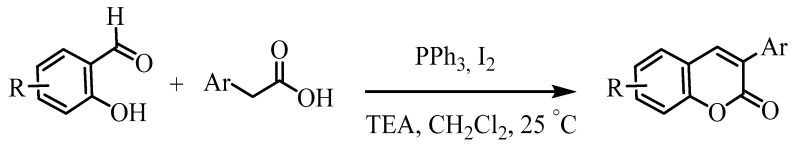
Synthetic route to access aryl-functionalized coumarins.

Once we prepared **3**, we turned our attention to the method reported by Phakhodee et al. for the synthesis of coumarins starting from substituted salicylaldehydes (Scheme 3) [33].

**Scheme 4:**
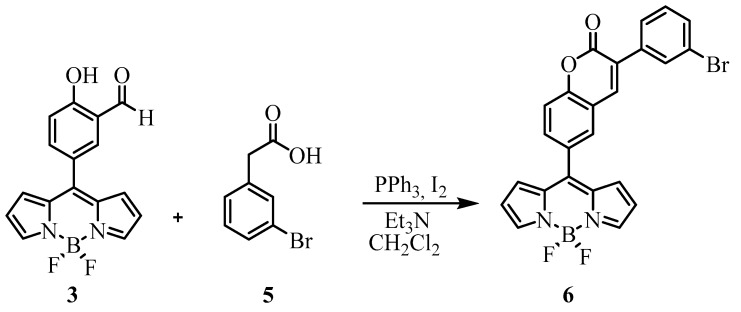
Reaction between BODIPY and 3-bromophenylacetic acid.

In this fashion, BODIPY **3** was reacted with 3-bromophenylacetic acid **5** (Scheme 4). The reaction was operationally very simple and was performed between 0 °C and room temperature under air. BODIPY-coumarin **6** precipitates and after filtration and crystallization, it was obtained with a 59% yield.

Next, we studied the scope and limitations of the functionalization of **6**, under the Suzuki cross-coupling standard conditions (Table 1).

The yields of the reactions ranged from good to excellent. The Suzuki reaction works efficiently regardless of whether electron-donating or electron-withdrawing boronic acids are used. Heteroarylboronic acids cross-coupled efficiently (entries 5, 7, and 10). Reaction with *p*-chlorophenylboronic acid (entry 11) gave a complex mixture without a major compound. Presumably, once the initial product is formed, the chlorine atom reacts further under the reaction conditions. An attempt to prepare a dimeric analogue using *p*-phenyldiboronic acid failed as well. No evidence for the formation of the desired product was observed by NMR of the crude material. Tetraphenylethene derivative **7i** was prepared in the hope that it would display aggregation-induced emission (AIE) [34,35,36], however, disappointingly, it did not.

### 2.2. Photophysical Properties

**Figure 2:**
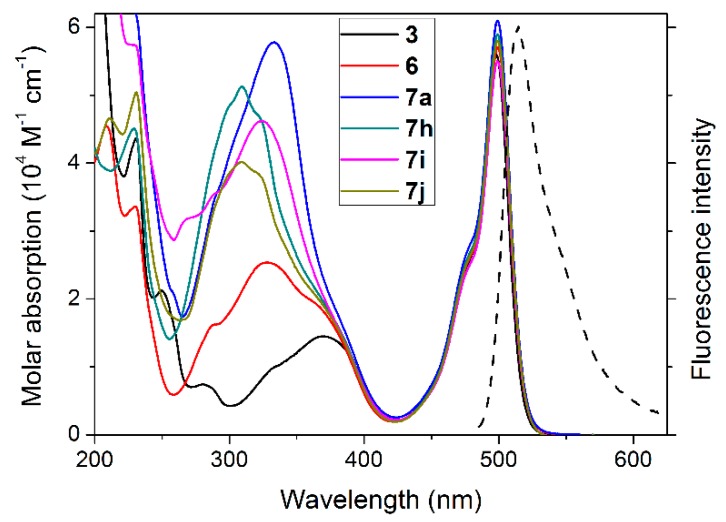
UV-Vis absorption and fluorescence (dashed line) of representative BODIPY-coumarin hybrids in diluted solution of cyclohexane. The reference BODIPY not bearing 8-coumarin **3** is also included for comparison. Note that the shape and position of the fluorescence spectra does not change nor with the kind of tethered coumarin neither the excitation wavelength (UV or Vis) owing to the ongoing intra-EET. The absorption spectra of the rest of the dyads are collected in Figure S1 in Supplementary Materials.

The spectroscopic properties of the BODIPY-coumarin hybrids in the visible spectral region are ruled by the spectral bands owned to the BODIPY subunit. Indeed, sharp absorption and fluorescence bands were registered at around 500 nm and 520 nm, respectively, regardless of the kind of coumarin appended at the *meso* position (Figure 2). Therefore, the coumarin subunit is electronically decoupled with the dipyrromethene backbone and the profile of the visible spectral bands of the dyads fully remained to those of the BODIPY alone. However, the presence of such moieties at the sterically unconstrained *meso* position has a deep impact on the fluorescence response. In fact, the fluorescence efficiency and lifetime harshly decreased due to the presence of the coumarin at an 8-position, yielding values lower than 5% and faster than 500 ps for all the tested compounds (Table S1 in Supplementary Materials). Such sudden enhancement of the non-radiative rate constants was attributed at first sight to the deactivation channels afforded by the free rotation of the 8-coumarin fragment directly linked to the BODIPY. Indeed, low fluorescence efficiencies have been reported for BODIPYs bearing unconstrained aryls at the said key *meso* position [37,38,39], as supported by the theoretically conducted potential energy curves, which highlight the key role on the photophysics of the conformational freedom around the linkage bond between the BODIPY and the 8-aryls [40,41,42,43].

Whereas no change was detected in the visible absorption and fluorescence, the UV absorption remarkably changed depending on the kind of coumarin placed at the *meso* position, in particular, on the aromatic functionalization added to the chromene core at 3-position (Figure 2 and Figure S1 in Supplementary Materials). The coumarin absorption band was detected at 325 nn, with its long-wavelength tail overlapped with the more energetic transitions of the BODIPY (S_0_→S_2_ and S_0_→S_3_, energetically close, and giving a broad and weak band placed at around 375 nm, see **3** in Figure 2). Nevertheless, in the dyads where the coumarin is functionalized with electron-rich groups, like triphenylamine (**7a**), stilbene (**7h** and **7i**) and benzothiophene (**7j**), a marked increase in the absorption within 275–375 nm was clearly recorded (up to 2-fold regarding to **6,** bearing the simplest coumarin unit, and up to 5 fold with respect to the BODIPY in **3**). Indeed, in these last dyads, the molar absorption of the band attributed to the modified coumarin became almost equal to the Vis band of the BODIPY. It is likely that these functionalizations of the chromene core with aromatic groups promote a more π-extended system of the coumarin. Strikingly, such spanning of the conjugation was not reflected in the ensuing pronounced bathochromic shift, but led to a marked enhancement of the absorption probability of the coumarin. It is noteworthy that the spectral profiles of all the compounds remained the same after prolonged UV irradiation or long aging times, evidencing their chemical stability and photostability.

**Figure 3:**
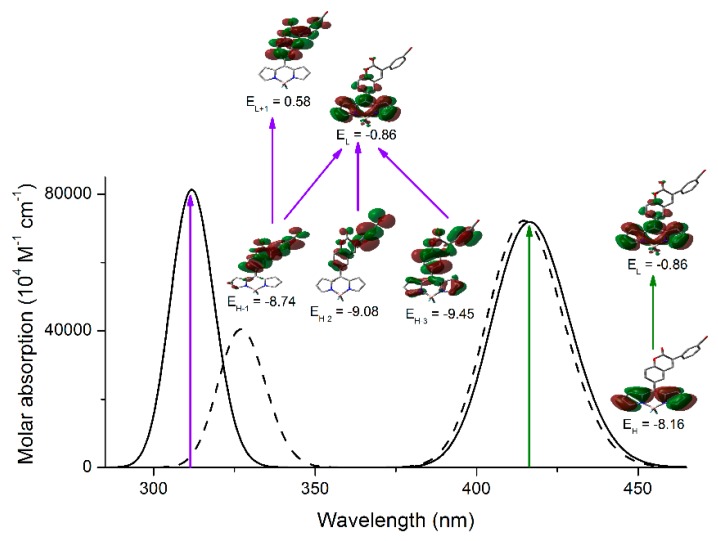
Predicted absorption spectrum (td wb97xd/6-311+g*) and main molecular orbitals and energies (in eV) involved in the electronic transitions for dyad **6**. The corresponding spectrum for reference compound **3** (dashed line) is added for comparison. The predicted absorption spectra and molecular orbitals for other representative dyads with π-extended coumarins are collected in Figure S2 in Supplementary Materials.

The computationally predicted absorption profiles matched the experimentally recorded ones and support the aforementioned assignment of the spectral bands and their trends with the functionalization of the coumarin (Figure 3 and Figure S2 in Supplementary Materials). The theoretical simulation of the energy gap for the band placed at the UV region was much accurate than for that located in the visible region. This is a typical drawback of the Time Dependent (TD-DFT) method; as the spectral band is shifted to lower energies, the method overestimates the energy gap [44,45]. The predicted visible absorption owned exclusively to the molecular orbitals of the BODIPY (HOMO→LUMO), whose position remained invariant with the type of appended 8-coumarin. Additionally, a UV band was predicted in the spectral region where the highest electronic transition of the BODIPY were placed (Figure 3), but with growing intensity when the coumarin is decorated with electron-rich groups (Figure 3 and Figure S2 in Supplementary Materials). The analysis of the molecular orbitals involved in such transition revealed that it was the consequence of many configurations. For instance, in dye **6,** the occupied orbitals were located preferentially at the coumarin moiety (HOMO-1 and -2), but with the electronic density shifted to the pendant phenyl (Figure 3), and eventually reaching the appended aromatic functionalization of such a ring in more complex coumarins. The overlapping with the highest transitions of the BODIPY can be clearly visualized in HOMO-3 (Figure 3), which is delocalized through the whole molecule, although the dipyrrin and the coumarin are electronically decoupled in the ground state. It is noticeable that the virtual orbitals involved in this UV transition are preferentially placed at the BODIPY (LUMO).

**Figure 4:**
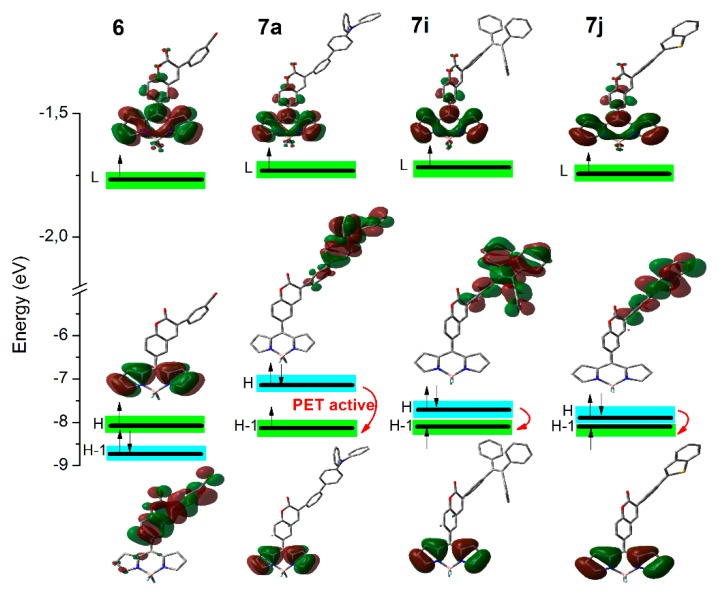
Main calculated molecular orbitals from the optimized geometries (wb97xd/6-311+g*) of the dyads with π-extended coumarins **7a, 7i** and **7j**, compared with those computed for the dyad bearing the simplest coumarin **6,** to illustrate the viability of the electron transfer upon selective excitation of the BODIPY in the former dyads.

Strikingly, just in those dyads bearing electron-rich groups (like the aforementioned triphenylamine, stilbene and benzothiophene), the predicted energetic ordering of the molecular orbitals suggests that they are able to switch on a reductive photoinduced electron transfer (PET) [46,47] pathway. In these dyes, the HOMO is located at the coumarin (**7a**, bearing a strong electron donor like triphenyalmine) rather than in the dipyrrin as expected. In other words, the energy of the highest occupied orbital of the coumarin falls between the energy gap of the frontier orbitals of the BODIPY. In the rest of dyads with less electron rich coumarins (like **6**), the frontier orbitals of the coumarin are placed up and down the orbitals responsible of the visible absorption of BODIPY, hence not interfering with them (Figure 4). As a matter of fact, the presence of electron donor triplenylamine **7a** at the coumarin raises the energy of the highest occupied orbital placed at the coumarin around 1.6 eV, being the dyad where the PET is more feasible (Figure 4). Thus, in dyads like **7a, 7i** or **7j**, after selective excitation of the BODIPY (HOMO-1→LUMO in this case), the electron-rich moieties can inject an electron into the BODIPY (a thermodynamically feasible hop), hampering the fluorescence deactivation. Such PET can be also anticipated from the analysis of the molecular orbitals involved in the UV absorption, since excitation implies transfer of electronic density from the coumarin to the dipyrrin core, supporting the electron donor nature of the 8-appended coumarins (Figure 3). This quenching pathway adds another non-radiative channel to the aforementioned internal conversion enhancement prompted by the 8-aryl free motion, explaining the recorded extremely low fluorescence efficiencies for these BODIPY-coumarin hybrids (Table S1 in Supplementary Materials).

Therefore, these dyads bearing π-extended coumarins (mainly **7a, 7h, 7i** and **7j**) improve the light harvesting efficiency of the BODIPY-coumarin hybrids, guaranteeing a better and broader collection of the incoming light to promote the ulterior energy transfer. Indeed, in all the dyads regardless of the excitation wavelength and the selectively excited subunit, just the visible emission from the BODIPY was recorded, without any sign of the emission from the coumarin (Figure 2). Thus, the intramolecular excitation energy transfer (intra-EET) from the donor coumarin to the acceptor BODIPY is highly efficient, although the fluorescence output is low owing to the said non-radiative pathways (conformational free rotation around the 8-position of the BODIPY and eventually PET).

**Figure 5:**
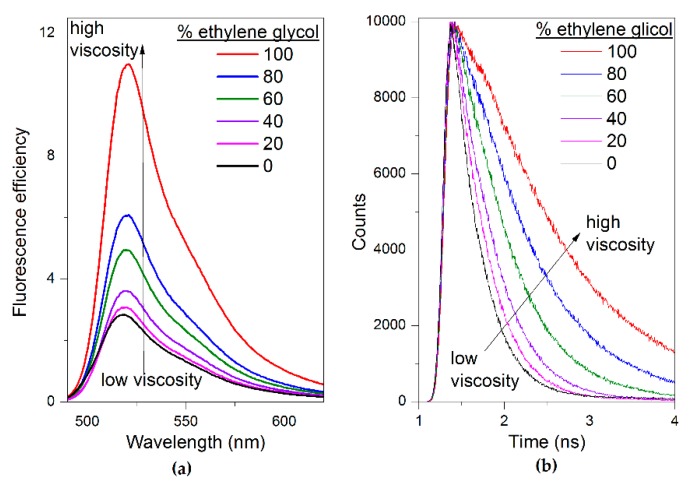
Evolution of the (**a**) fluorescence spectra (scaled by the fluorescence quantum yield) and (**b**) decay curves of dyad **6** with the content of ethylene glycol in diluted ethanolic solutions.

Non-fluorescent dyes owing to the said conformational freedom (including dyads, such as coumarin-rhodamine) [48,49], are being currently applied as molecular rotors to monitor the microviscosity of the surrounding environment, even in the cellular media [50,51]. Accordingly, we hypothesized that, in the herein reported BODIPY-coumarin dyads, as the viscosity of the media increases, the free rotation of the 8-aryl should be hampered, and consequently the fluorescence quantum yield should increase and the lifetime lengthen, with this last property being very sensitive to such environmental property in view of the reported results in the bibliography. Therefore, we have tested the performance of dyad **6**, as a representative compound of the herein reported BODIPY-coumarin dyads not undergoing PET, as a molecular rotor to monitor the viscosity of the surrounding media. To this aim, firstly we have measured its photophysics in a viscous solvent like ethylene glycol. Successfully, the fluorescence quantum yield and lifetime in this viscous solvent were clearly higher and longer, respectively, (up to around 0.15 and 1 ns, in comparison with the rest of solvents in Table S1 in Supplementary Materials, with values lower than 0.05 and 450 ps, respectively). Such improvement of the fluorescence emission is nicely supported by the recorded fluorescence spectra and decay curves in media with controlled viscosity by means of ethanol/ethylene glycol mixtures (Figure 5). As the viscosity of the media progressively increases the emission spectra becomes more intense and the decay curves slower, suggesting that the free motion of the 8-aryl group is hampered and consequently the associated non-radiative relaxation is also hindered.

**Figure 6:**
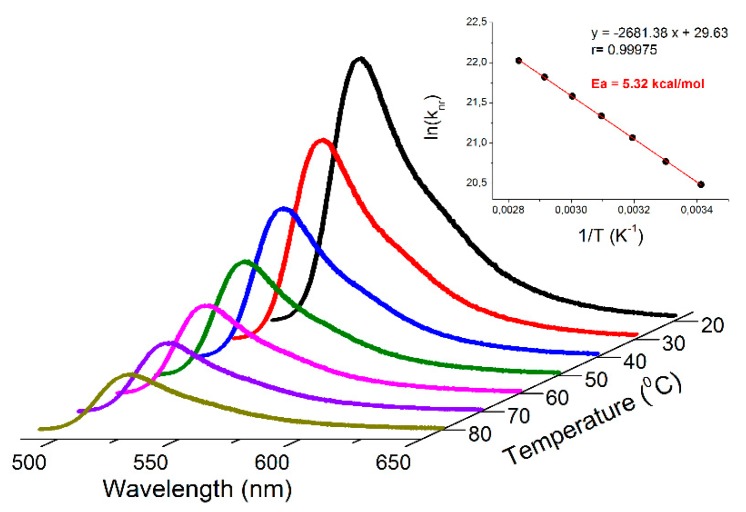
Evolution of the fluorescence spectra of dyad **6** with the temperature in ethylene glycol. The corresponding Arrhenius plot for the non-radiative rate (k_nr_) constant is also enclosed. The k_nr_ data at each temperature was calculated after checking that the absorption spectra are the same regardless of the temperature, and assuming that the radiative rate constant (k_fl_) does not change with the temperature. In other words, the loss of fluorescence signal upon heating is due solely to an increase of the internal conversion related to the 8-aryl motion.

Such key influence of the viscosity in the fluorescence response can be also visualized following the evolution of the emission intensity with the temperature in ethylene glycol. Indeed, as the temperature increases, there is more energy available to rotate the 8-aryl and to overcome the impediment afforded by the environmental viscosity. Thus, a heating of the solution progressively decreased the emission efficiency owing to the discussed enhancement of the internal conversion processes (Figure 6). In fact, an activation energy of around 5.3 kcal/mol has been calculated from the evolution of the non-radiative rate, constant with the temperature in ethylene glycol (Figure 6).

## 3. Materials and Methods

### 3.1. Materials

Starting 8-methylthioBODIPY, CuTC, tri(2-furyl)phosphine, and boronic acids are commercially available. Solvents were dried and distilled before use.

### 3.2. General Procedure for the Suzuki Reaction

In a reaction tube under N_2_, we dissolved **6** (1.0 equiv), the corresponding boronic acid (2.0 equiv), Pd(OAc)_2_ (5 mol%), S-Phos (15 mol%), Na_2_CO_3_ (2.0 equiv) in a mixture toluene/H_2_O (4:1, 2.5 mL). The reaction was heated at 90 °C until the starting material was consumed as indicated by thin-layer chromatography (TLC), cooled to room temperature, and then water was added. Then it was extracted with ethyl acetate (3 × 10 mL), washed with brine, dried over MgSO_4_, filtered and evaporated to dryness. The crude was filtered through a short silica gel column, and eluted with dichloromethane (DCM). The product was crystalized using DCM/petroleum ether.

### 3.3. Synthesis and Characterization

^1.^H and ^13^C Nuclear magnetic Resonance (NMR) spectra (collected in the Supplementary Materials) were recorded on a Bruker (Billerica, MA, US) Avance III HD 400 (^1^H, 400MHz; ^13^C 100 MHz) or Bruker Ultrashield 500 (^1^H, 500 Mhz; ^13^C 125 MHZ) in deuteriochloroform (CDCl_3_), with either tetramethylsilane (TMS) (0.00 ppm ^1^H, 0.00 ppm ^13^C), chloroform (7.26 ppm ^1^H, 77.00 ppm ^13^C). Data are reported in the following order: chemical shift in ppm, multiplicities (br (broadened), s (singlet), d (doublet), t (triplet), q (quartet), m (multiplet), exch (exchangeable), app (apparent)), coupling constants, *J* (Hz) and integration. Infrared spectra were recorded on a Perkin Elmer (Waltham, MA, US) Spectrum 100 Fourier-transform infrared (FTIR) spectrophotometer. Peaks are reported (cm^−^^1^) with the following relative intensities: s (strong, 67–100%), m (medium, 40–67%), and w (weak, 20–40%). Melting points are not corrected. TLC was conducted in Silica gel on TLC Al foils. Detection was done by UV light (254 or 365 nm). High-resolution mass spectrometry (HRMS) samples were determined on a MaXis Impact ESI-QTOF-MS (Bruker Daltonics) by electrospray ionization in positive mode (ESI+) and recorded via the time of fly (TOF) method.

The corresponding reaction conditions for each compound as well as their characterization data are detailed in the Supplementary Materials.

### 3.4. Spectroscopic Measurements

The photophysical properties were registered using quartz cuvettes with optical pathways of 1 cm in diluted solutions (around 2 × 10^−6^ M), prepared by adding the corresponding solvent to the residue from the adequate amount of a concentrated stock solution in acetone, after vacuum evaporation of this solvent. Ultraviolet-visible (UV-vis) absorption and fluorescence spectra were recorded on a Varian model CARY 4E spectrophotometer (Agilent Technologies, Santa Clara, CA, US), and an Edinburgh Instruments (Livingston, England) spectrofluorimeter (model FLSP920), respectively. Fluorescence quantum yields (ϕ) were obtained using PM546 as a reference (Exciton, ϕ^r^ = 0.85 in ethanol). Radiative decay curves were registered with the time correlated single-photon counting technique, as implemented in the aforementioned spectrofluorimeter. Fluorescence emission was monitored at the maximum emission wavelength by using a microchannel plate detector (Hamamatsu C4878) of picosecond time-resolution (20 ps), after excitation with a Fianium pulsed laser (time resolution of around 150 picoseconds). The fluorescence lifetime (τ) was obtained after the deconvolution of the instrumental response signal from the recorded decay curves by means of an iterative method. The goodness of the exponential fit was controlled by statistical parameters (chi-square) and the analysis of the residuals. Radiative (k_fl_) and non-radiative (k_nr_) rate constants were calculated as follows: k_fl_ = ϕ/τ; k_nr_ = (1 − ϕ)/τ.

### 3.5. Computational Simulations

Ground state energy minimizations were performed using a functional range-separated hybrid wb97xd within the Density Functional Theory (DFT), using the triple valence basis set with a polarization and a diffuse function (6-311+g*). The optimized geometries were taken as a true energy minimum using frequency calculations (no negative frequencies). The conformational search around the linkage bond between the coumarin fragment and the BODIPY at 8-positions suggests that the aforementioned geometry corresponds to the most stable conformer. The absorption profile was simulated with the Time Dependent (TD-DFT) method using the same calculation level and basis set. The Polarizable Continuum Model (PCM) was considered to have a solvent effect (cyclohexane) in all the calculations. All the calculations were performed in Gaussian 16, using the “arina” computational resources provided by the UPV-EHU.

## 4. Conclusions

Salicylaldehyde was efficiently functionalized with a BODIPY unit via a Liebeskind-Srogl cross-coupling reaction. In a first example of the application of **3** as a building block, a *meta*-bromophenyl BODIPY-coumarin was prepared from which 10 novel analogues were attained using the Suzuki reaction. The addition of coumarins, functionalized with aromatic moieties, to the *meso* position of BODIPYs is a suitable synthetically accessible strategy to ameliorate the light harvesting ability of BODIPY-coumarin hybrids. The proper selection of the functional aromatic groups to extend the π-system of the chromene core, enables the enhancement of the absorption probability at the UV-blue region, providing a more efficient and broader light collection for the subsequent excitation energy transfer to the BODIPY. The low fluorescence response of the herein reported dyads is attributed to the conformational freedom of the coumarin around the key *meso* position and the activation of electron transfer processes when electron-rich groups are tethered at the coumarin subunit.

However, the detrimental impact of the conformational flexibility on the fluorescence response of the BODIPY-coumarin hybrids paves the way to apply them as molecular rotors for the monitorization of the environmental microviscosity. In fact, in those dyads not undergoing PET, the fluorescence efficiency and lifetime progressively increases and lengthens, respectively, with the viscosity of the media. Therefore, these BODIPY-coumarin dyads behave as versatile molecular rotors to quantify the viscosity following the changes of the fluorescence signatures upon excitation almost along the whole UV-yellow spectral region, owing to their broadband light harvesting and ensuing energy transfer.

## Figures and Tables

**Table 1:**
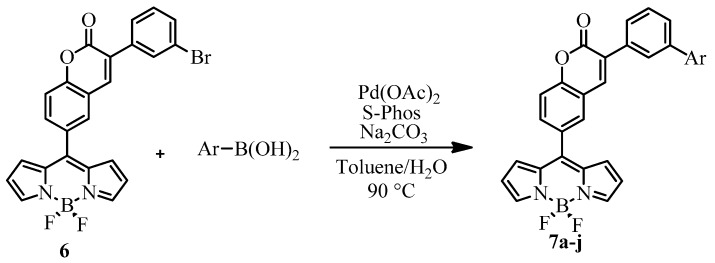
Scope and limitations of the Suzuki cross-coupling reaction of BODIPI-coumarin **6**.^a.^

Entry	Ar-	Reaction time (h)	% yield ^b^	cpd
1	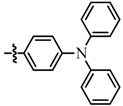	21	75	**7a**
2	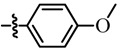	16	85	**7b**
3	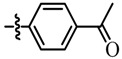	19	85	**7c**
4	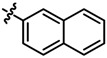	20	68	**7d**
5	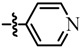	20	76	**7e**
6	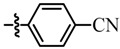	17	62	**7f**
7	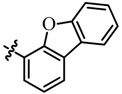	18	68	**7g**
8	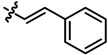	16	93	**7h**
9	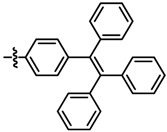	6.5	82	**7i**
10	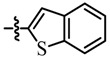	7	77	**7j**
11	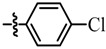	-	- ^c^	**7k**
12	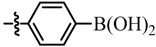	-	- ^d^	**7l**

^a^ Conditions: **6** (1 equiv), boronic acid (2.0 equiv), Pd(OAc)_2_ (5 mol%), S-Phos (15 mol%), Na_2_CO_3_ (2.0 equiv.) /H_2_O (4:1) at 90 °C. ^b^ Isolated yield. ^c^ Inseparable complex mixture. ^d^ The expected product was not detected by NMR.

## Supplementary Materials

The following are available online; synthetic details and characterization data (IR, NMR, HRMS) of each compound, ^1^H and ^13^C-NMR spectra, photophysical data (Table S1), absorption spectra (Figure S1) and computed absorption spectra (Figure S2).
