# Supplementary material for: Ready Access to Molecular Rotors Based on Boron Dipyrromethene Dyes-Coumarin Dyads Featuring Broadband Absorption
Source: Molecules. 2020 Feb 12;25(4):781. doi: 10.3390/molecules25040781 (PMC7070740; doi:10.3390/molecules25040781)
Supplement: Supplementary file 1 [file molecules-25-00781-s001.pdf]

Supplementary Materials

Ready access to molecular rotors based on BODIPY-coumarin dyads featuring broadband absorption

Ernesto Enríquez-Palacios [1], Teresa Arbeloa [2], Jorge Bañuelos [2,*], Claudia I. Bautista-Hernández [1], José G. Becerra-González [1], Iñigo López Arbeloa [2], Eduardo Peña-Cabrera [1,*].

[1] Departamento de Química, Universidad de Guanajuato. Noria Alta S/N. Guanajuato, Gto. 36050, Mexico. e.enriquezpalacios@ugtomx.com (E.E.-P); cla_isb_6@hotmail.com (C.I.B.-H.); jg.becerragonzalez@ugto.mx (J.G.B.-G.); eduardop@ugto.mx (E.P.-C.)

[2] Departamento de Química Física. Universidad del País Vasco-EHU, Apartado 644, 48080, Bilbao, Spain. teresa.arbeloa@ehu.es (T.A.); jorge.banuelos@ehu.es (J. B.); inigo.lopezarbeloa@ehu.es (I.L.A.)

* Correspondence: jorge.banuelos@ehu.es (J.B); eduardop@ugto.mx (E.P.-C.)

Synthesis details and characterization data S2

^{1}H and ^{13}C-NMR spectra S9

Table 1. Photophysical data S33

Figure S1. Absorption spectra S35

Figure S2. Computed absorption spectra S36

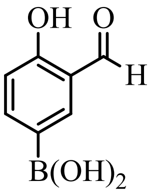

2

Synthesis of 2. In a reaction tube under N_2, were dissolved $Pd(OAc)_2$ (2.0%, $15x10^{-3}$ mmol), X-Phos (6.0%, $48x10^{-3}$ mmol), KOAc (2.0 equiv, 1.5 mmol), in ethanol (0.1M). The reaction mixture was degassed and heated 30 minutes at 70 ℃. To the reaction was added **4** (1.0 equiv, 0.75 mmol) and $B_2(OH)_4$ (2.0 equiv, 1.49 mmol). The reaction was heated at 70 ℃ until complete disappearance of starting material as indicated by TLC monitoring. The reaction was cooled to room temperature, filtered through a celite (eluting with ethyl acetate) pad and the solvent was evaporated to dryness. The product was purified by silica gel column (3-10% MeOH/hexanes), to afford **2** (72%) as white solid. ^{1}H NMR (500 MHz, d_6-DMSO): δ 10.85 (s, 1H), 10.24 (s, 1H), 8.15 (d, J = 1.3 Hz, 1H), 7.91 (dd, J = 8.3, 1.5 Hz, 1H), 6.95 (d, J = 8.2 Hz, 1H).

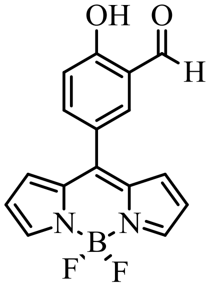

3

Synthesis of 3. In a reaction tube under N_2, were dissolved 8-methythioBODIPY **1** (1 equiv, 0.210 mmol), **2** (2.5 equiv, 0.525 mmol), $Pd_2(dba)_3$ (2.5%, $5.2x10^{-3}$ mmol), tri(2-furyl)phosphine (7.5%, 0.015 mmol), CuTC (2.5 equiv, 0.630 mmol) in THF (0.03M). The reaction was heated at 55 ℃ until complete disappearance of starting material as indicated by TLC monitoring. The reaction was cooled to room temperature, and then the solvent was evaporated to dryness. The product was purified by passing crude through a short silica gel column eluting with DCM, and crystallization (DCM/petroleum ether), to afford **3** (75%) as green crystals. TLC (1:3 AcOEt/hexanes, R_f = 0.30); mp 179-180 ℃; IR (KBr, cm^{-1}): 3125 (m), 1659 (s), 1547 (s), 1477 (m), 1412 (s), 1391 (s), 1352 (w) 1291 (m), 1260 (s), 1219 (s), 1179 (w), 1117 (s), 1080 (s), 993 (m), 945 (m), 771 (m), 747 (m), 721 (m), 701 (m), 639 (w), 627 (w); ^{1}H NMR (400 MHz, $CDCl_3$) δ 11.33 (s, 1H), 9.98 (s, 1H), 7.95 (s, 2H), 7.81 (d, J = 2.0 Hz, 1H), 7.75 (dd, J = 8.6, 2.1 Hz, 1H), 7.18 (d, J = 8.6 Hz, 1H), 6.93 (d, J = 4.1 Hz, 2H), 6.58 (d, J = 3.7 Hz, 2H); ^{13}C NMR (126 MHz, $CDCl_3$) δ 196.2, 163.7, 145.3, 144.6, 138.6, 136.0, 134.9, 131.1, 125.8, 120.5, 119.0, 118.6. HRMS (ESI+) m/z calcd for $C_{16}H_{11}BF_2N_2O_2Na$ $[M+Na]^+$ 335.0777. Found 335.0781.

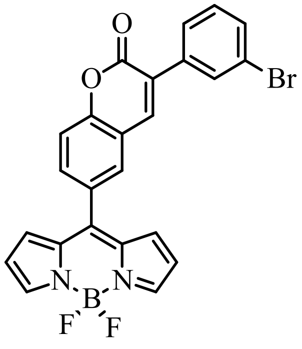

6

Synthesis of 6. In a one neck round bottom flask was dissolved PPh_3 (1.4 equiv, 0.490 mmol) in DCM, the solution was cooled to 0 ℃, iodine (1.4 equiv, 0.490 mmol) was added and the mixture was stirred for 30 min, then **3** (1.0 equiv, 0.350 mmol), **5** (1.1 equiv, 0.385 mmol) were added, finally Et_3N (5.0 equiv, 1.60 mmol) added dropwise. After that the mixture was allowed to warm up to r.t. and stirred during 1 h. The crude was filtered through short silica gel column using DCM and crystalized using DCM/petroleum ether to obtain product as a dark green crystals in a 59% yield. TLC (1:3 AcOEt/Hexane, R_f: 0.38); mp 214-215 ℃; IR (KBr, cm^{-1}): 3111 (w), 1728 (s), 1607 (m), 1554 (s), 1478 (m), 1412 (s), 1386 (s) 1355 (w), 1261 (s), 1202 (s), 1114 (s), 1078 (s), 1048 (m), 995 (m), 957 (m), 840 (w), 777 (m), 760 (m), 745 (m), 728 (w), 690 (w), 590 (w); ^1H NMR (500 MHz, $CDCl_3$) δ 7.98 (s, 2H), 7.89 (s, 1H), 7.88 (t, *J* = 1.7 Hz, 1H), 7.78 (d, *J* = 1.9 Hz, 1H), 7.75 (dd, *J* = 8.5, 1.9 Hz, 1H), 7.68 (d, *J* = 7.8 Hz, 1H), 7.57 (d, *J* = 8.0 Hz, 1H), 7.54 (d, *J* = 8.5 Hz, 1H), 7.35 (t, *J* = 7.9 Hz, 1H), 6.92 (d, *J* = 4.1 Hz, 1H), 6.59 (d, *J* = 3.3 Hz, 1H); ^{13}C NMR (126 MHz, $CDCl_3$) δ 159.4, 154.9, 144.9, 144.8, 139.4, 136.0, 134.9, 133.4, 132.3, 131.4, 131.2, 130.3, 130.1, 129.9, 128.4, 127.2, 122.6, 119.5, 119.1, 119.1, 117.0. HRMS (ESI+) *m/z* calcd for $C_{24}H_{15}BBrF_2N_2O_2$ $[M+H]^+$ 493.0359. Found 493.0357.

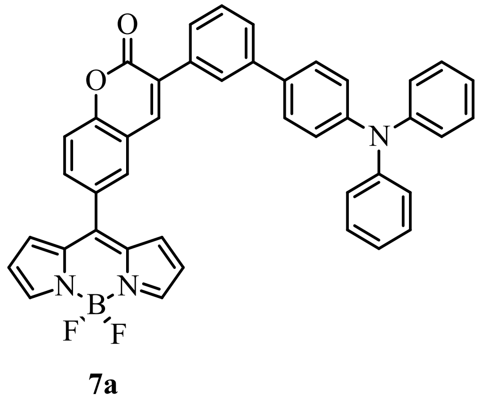

7a

Synthesis of 7a. According to general procedure. **6** (15 mg, 0.030 mmol), (diphenylamino)phenyl boronic acid (17.7 mg, 0.060 mmol), $Pd(OAc)_2$ (0.4 mg, $1.5x10^{-3}$ mmol), S-Phos (1.9 mg, $4.6x10^{-3}$ mmol), Na_2CO_3 (6.5 mg, 0.060 mmol). Reaction time 21 h; mp: 159-160 ℃; R_f: 0. (AcOEt/Hexane, 1:3); 75% yield as orange solid; IR (KBr, cm^{-1}): 3035 (w), 1736 (s), 1592 (m), 1555 (s), 1485 (m), 1412 (m), 1386 (s), 1355 (w), 1261 (s), 1200 (w), 1114 (s), 1078 (s), 996 (m), 957 (m), 836 (w), 755 (m), 742 (w), 697 (m), 511 (w); ^1H NMR (500 MHz, DMSO) δ 8.48 (s, 1H), 8.18 (s, 2H), 8.12 (s, 1H), 7.98 (s, 1H), 7.90 (d, *J* = 8.5 Hz, 1H), 7.71 (d, *J* = 7.7 Hz, 1H), 7.69 (d, *J* = 7.8 Hz, 1H), 7.66 - 7.63 (m, 3H), 7.54 (t, *J* = 7.7 Hz, 1H), 7.33 – 7.30 (m, 4H), 7.12 (d, *J*

= 3.6 Hz, 1H), 7.07 - 7.05 (m, 8H), 6.72 (d, *J* = 2.3 Hz, 1H); ^{13}C NMR (126 MHz, DMSO) δ 159.3, 154.5, 147.0, 146.9, 145.2, 145.1, 140.4, 139.8, 135.0, 134.2, 133.7, 133.6, 131.9, 131.0, 129.6, 129.1, 128.9, 127.8, 127.7, 127.1, 126.7, 126.4, 124.1, 123.4, 123.3, 119.7, 119.5, 116.5. HRMS (ESI+) *m/z* calcd for $C_{42}H_{28}BF_2N_3O_2$ $[M]^+$ 655.2244. Found 655.2263.

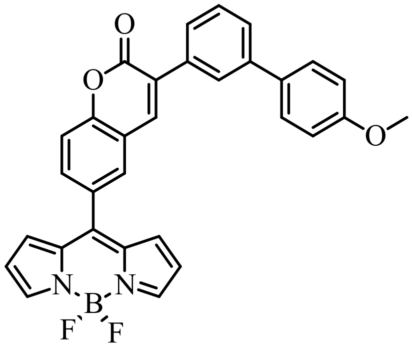

7b

Synthesis of 7b. According to general procedure. **6** (15 mg, 0.030 mmol), (**4-methoxyphenyl)**boronic acid (9.3 mg, 0.060 mmol), $Pd(OAc)_2$ (0.4 mg, 1.5x10^{-3} mmol), S-Phos (1.9 mg, 4.6x10^{-3} mmol), Na_2CO_3 (6.5 mg, 0.060 mmol). Reaction time 16 h; mp: 240-241 ℃; R_f: 0.45 (AcOEt/Hexane, 1:3); 85% yield as dark green crystals; IR (KBr, cm^{-1}): 3118 (w), 2937 (w), 2837 (w), 1721 (s), 1606 (m), 1543 (s), 1353 (w), 1256 (s), 1205 (m), 1114 (s), 1080 (s), 1052 (m), 993 (m), 959 (m), 908 (w), 848 (m), 834 (m), 798 (m), 783 (m), 749 (m), 697 (w), 571 (w); ^{1}H NMR (500 MHz, $CDCl_3$) δ 7.97 (s, 2H), 7.92 (s, 1H), 7.88 (t, *J* = 1.6 Hz, 1H), 7.77 (d, *J* = 2.0 Hz, 1H), 7.73 (dd, *J* = 8.5, 2.1 Hz, 1H), 7.66 (d, *J* = 7.8 Hz, 1H), 7.62 (d, *J* = 7.9 Hz, 1H), 7.57 (d, *J* = 8.7 Hz, 2H), 7.52 (m, 2H), 7.00 (d, *J* = 8.7 Hz, 2H), 6.93 (d, *J* = 4.2 Hz, 2H), 6.58 (d, *J* = 2.9 Hz, 2H); ^{13}C NMR (126 MHz, $CDCl_3$) δ 159.8, 159.5, 154.9, 145.0, 144.8, 141.4, 138.8, 134.9, 134.6, 133.1, 133.0, 131.3, 130.2, 129.9, 129.8, 129.0, 128.3, 127.7, 127.0, 126.8, 119.8, 119.0, 116.9, 114.3, 55.4. HRMS (ESI+) *m/z* calcd for $C_{31}H_{22}BF_2N_2O_3$ $[M+H]^+$ 519.1691. Found 519.1694.

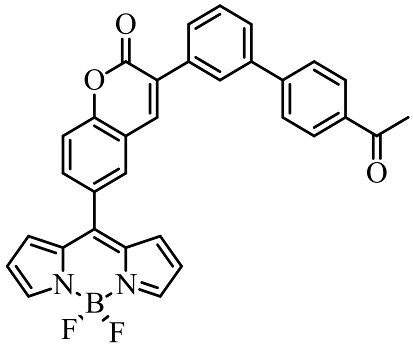

7c

Synthesis of 7c. According to general procedure. **6** (15 mg, 0.030 mmol), 4-acetylphenylboronic acid (10 mg, 0.060 mmol), $Pd(OAc)_2$ (0.4 mg, 1.5x10^{-3} mmol), S-Phos (1.9 mg, 4.6x10^{-3} mmol), Na_2CO_3 (6.5 mg, 0.060 mmol). Reaction time 19 h; mp: 162-163 ℃; R_f: 0.22 (AcOEt/Hexane, 1:3); 85% yield as dark green crystals; IR (KBr, cm^{-1}): 3130 (w), 3064 (w), 2926 (w), 1735 (s), 1682 (s), 1605 (s), 1551 (s), 1480 (m), 1413 (s), 1386 (s), 1355 (w), 1261 (s), 1200 (m), 1114 (s), 1081 (s), 1046 (m), 995 (m), 996 (s), 907 (w), 845 (w), 799 (w), 774 (w), 744 (w), 699 (w), 594 (w); ^{1}H NMR (500 MHz, $CDCl_3$) δ 8.05 (d, *J* = 8.4 Hz, 2H), 8.00 – 7.94 (m, 4H), 7.79 (d, *J* = 2.0 Hz, 1H), 7.77 – 7.68 (m, 5H), 7.60 – 7.53

(m, 1H), 7.58 (t, *J* = 7.8 Hz, 1H), 7.55 (d, *J* = 8.5 Hz, 1H), 6.58 (d, *J* = 3.0 Hz, 2H), 2.64 (s, 3H); ^{13}C NMR (126 MHz, $CDCl_3$) δ 197.6, 159.7, 154.9, 145.1, 144.9, 144.9, 140.4, 139.1, 136.2, 134.9, 133.2, 131.2, 130.3, 129.9, 129.6, 129.3, 129.0, 128.3, 128.2, 127.6, 127.4, 119.7, 119.0, 116.9, 26.7. HRMS (ESI+) *m/z* calcd for $C_{32}H_{22}BF_2N_2O_3$ $[M+H]^+$ 531.1692. Found 531.1694.

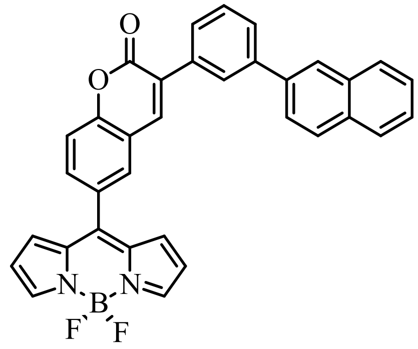

7d

Synthesis of 7d. According to general procedure. **6** (15 mg, 0.030 mmol), 2-naphthaleneboronic acid (10.5 mg, 0.060 mmol), $Pd(OAc)_2$ (0.4 mg, $1.5x10^{-3}$ mmol), S-Phos (1.9 mg, $4.6x10^{-3}$ mmol), Na_2CO_3 (6.5 mg, 0.060 mmol). Reaction time 20 h; mp: 209-210 ℃; R_f: 0.25 (AcOEt/Hexane, 1:3); 68% yield as dark green crystals; IR (KBr, cm^{-1}): 3107 (w), 3055 (w), 1728 (s), 1605 (w), 1551 (s), 1482 (m), 1412 (s), 1385 (s), 1354 (w), 1259 (s), 1201 (m), 1111 (s), 1081 (s), 996 (m), 958 (m), 859 (w), 801 (w), 780 (w), 746 (w), 700 (w), 590 (w), 479 (w); ^{1}H NMR (500 MHz, $CDCl_3$) δ 8.08 (s, 1H), 8.05 (s, 1H), 7.98 (s, 2H), 7.95 – 7.85 (m, 4H), 7.81 – 7.70 (m, 5H), 7.58 (t, *J* = 7.7 Hz, 1H), 7.55 – 7.47 (m, 3H), 6.93 (d, *J* = 4.1 Hz, 2H), 6.58 (d, *J* = 2.9 Hz, 1H); ^{13}C NMR (126 MHz, $CDCl_3$) δ 159.9, 154.9, 145.0, 144.8, 141.7, 139.0, 137.9, 134.9, 134.7, 133.6, 133.1, 132.8, 131.3, 130.2, 129.9, 129.2, 128.6, 128.4, 128.2, 127.7, 127.7, 127.5, 126.5, 126.2, 126.1, 125.5, 119.8, 119.0, 116.9. HRMS (ESI+) *m/z* calcd for $C_{34}H_{22}BF_2N_2O_2$ $[M+H]^+$ 539.1743. Found 539.1741.

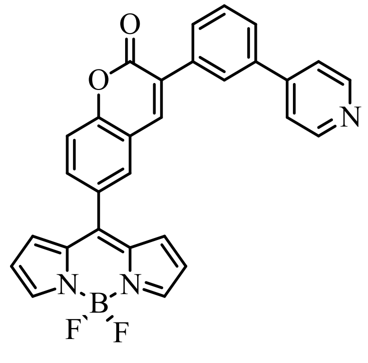

7e

Synthesis of 7e. According to general procedure. **6** (15 mg, 0.030 mmol), **4-pyridyl**boronic acid (7.5 mg, 0.060 mmol), $Pd(OAc)_2$ (0.4 mg, $1.5x10^{-3}$ mmol), S-Phos (1.9 mg, $4.6x10^{-3}$ mmol), Na_2CO_3 (6.5 mg, 0.060 mmol). Reaction time 20 h; mp: 143-144 ℃; R_f: 0.20 (AcOEt/Hexane, 4:6); 76% yield as orange solid; IR (KBr, cm^{-1}): 3105 (w), 1731 (s), 1596 (w), 1550 (s), 1479 (m), 1412 (s), 1386 (s), 1355 (w), 1260 (s), 1202 (m), 1114 (s), 1079 (s), 995 (m), 956 (m), 908 (w), 830 (w), 798 (w), 777 (w), 742 (w), 698 (w), 579 (w); ^{1}H NMR (500 MHz, DMSO) δ 8.67 (s,

2H), 8.52 (s, 1H), 8.18 (s, 2H), 8.13 (s, 2H), 7.93 (d, *J* = 8.5 Hz, 1H), 7.86 (d, *J* = 7.5 Hz, 2H), 7.77 (d, *J* = 4.5 Hz, 2H), 7.67 (d, *J* = 8.6 Hz, 1H), 7.64 (t, *J* = 7.8 Hz, 1H), 7.12 (d, *J* = 3.8 Hz, 2H), 6.72 (d, *J* = 3.7 Hz, 2H); ^{13}C NMR (126 MHz, DMSO) δ 159.8, 155.1, 150.8, 147.2, 145.6, 141.3, 137.9, 135.8, 134.7, 134.2, 132.4, 131.5, 129.8, 129.7, 129.6, 127.8, 127.8, 127.4, 121.9, 120.1, 120.0, 117.0. HRMS (ESI+) *m/z* calcd for $C_{29}H_{19}BF_2N_3O_2$ $[M+H]^+$ 490.1538. Found 490.1544.

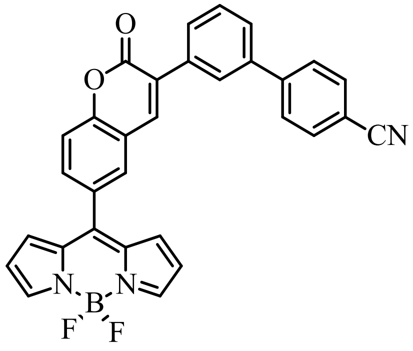

7f

Synthesis of 7f. According to general procedure. **6** (15 mg, 0.030 mmol), 4-cyanophenylboronic acid (9.0 mg, 0.060 mmol), $Pd(OAc)_2$ (0.4 mg, $1.5x10^{-3}$ mmol), S-Phos (1.9 mg, $4.6x10^{-3}$ mmol), Na_2CO_3 (6.5 mg, 0.060 mmol). Reaction time 17 h; mp: 150-151 ℃; R_f: 0.25 (AcOEt/Hexane, 1:3); 62% yield as dark green crystals**;** IR (KBr, cm^{-1}): 3068 (w), 2224 (s), 1732 (s), 1604 (m), 1542 (s), 1481 (m), 1412 (s), 1386 (s), 1352 (w), 1258 (s), 1204 (m), 1116 (s), 1087 (s), 1073 (s), 1048 (m), 997 (m), 958 (m), 908 (w), 849 (w), 800 (m), 777 (m), 746 (w), 697 (w), 643 (w), 579 (w); ^{1}H NMR (500 MHz, $CDCl_3$) δ 7.99 (s, 2H), 7.97 (s, 1H), 7.95 (s, 1H), 7.79 (d, *J* = 1.9 Hz, 1H), 7.78 – 7.73 (m, 6H), 7.67 (d, *J* = 7.9 Hz, 1H), 7.60 (t, *J* = 7.7 Hz, 1H), 7.57 (d, *J* = 8.5 Hz, 1H), 6.92 (d, *J* = 4.1 Hz, 2H), 6.59 (d, *J* = 3.1 Hz, 2H); ^{13}C NMR (126 MHz, $CDCl_3$) δ 159.7, 154.9, 145.0, 144.9, 139.7, 139.2, 135.1, 134.9, 133.3, 132.7, 131.2, 130.3, 129.8, 129.5, 129.4, 128.7, 128.2, 127.9, 127.6, 119.6, 119.0, 118.8, 117.0, 111.4. HRMS (ESI+) *m/z* calcd for $C_{31}H_{19}BF_2N_3O_2$ $[M+H]^+$ 514.1538. Found 514.1536.

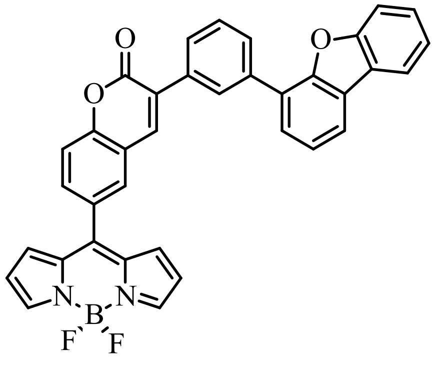

7g

Synthesis of 7g. According to general procedure. **6** (15 mg, 0.030 mmol), 4-(dibenzofuranyl)boronic acid (13.0 mg, 0.060 mmol), $Pd(OAc)_2$ (0.4 mg, $1.5x10^{-3}$ mmol), S-Phos (1.9 mg, $4.6x10^{-3}$ mmol), Na_2CO_3 (6.5 mg, 0.060 mmol). Reaction time 18 h; mp: 203-204 ℃; R_f: 0.42 (AcOEt/Hexane, 1:3); 68% yield as orange solid; IR (KBr, cm^{-1}): 3138 (w), 3055 (w), 1735 (s), 1605 (w),

1551 (s), 1480 (w), 1451 (w), 1413 (s), 1387 (s), 1354 (w), 1260 (s), 1190 (s), 1114 (s), 1080 (s), 1049 (w), 996 (s), 959 (s), 908 (w), 837 (w), 745 (s), 696 (w), 590 (w); ^{1}H NMR (500 MHz, $CDCl_3$) δ 8.23 (s, 1H), 8.01 – 7.93 (m, 6H), 7.80 (d, *J* = 7.8 Hz, 1H), 7.76 (d, *J* = 1.9 Hz, 1H), 7.72 (dd, *J* = 8.5, 2.0 Hz, 1H), 7.67 – 7.58 (m, 3H), 7.53 (d, *J* = 8.5 Hz, 1H), 7.49 – 7.42 (m, 2H), 7.37 (t, *J* = 7.5 Hz, 1H), 6.92 (d, *J* = 4.1 Hz, 2H), 6.57 (d, *J* = 2.9 Hz, 2H); ^{13}C NMR (126 MHz, $CDCl_3$) δ 159.8, 156.2, 154.9, 153.3, 145.0, 144.8, 139.0, 137.0, 134.9, 134.5, 133.0, 131.3, 130.2, 129.9, 129.8, 128.9, 128.9, 128.0, 127.4, 126.9, 125.2, 125.0, 124.1, 123.3, 122.9, 120.8, 120.1, 119.8, 119.0, 116.9, 111.9. HRMS (ESI+) *m/z* calcd for $C_{36}H_{22}BF_2N_2O_3$ $[M+H]^+$ 579.1692. Found 579.1693.

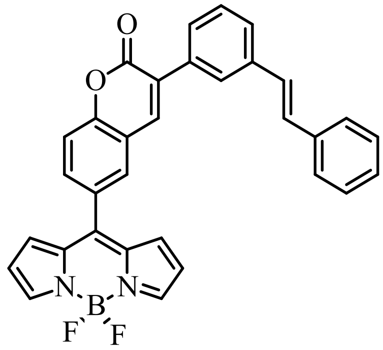

7h

Synthesis of 7h. According to general procedure. **6** (15 mg, 0.030 mmol), *trans*-2-Phenylvinylboronic acid, $Pd(OAc)_2$ (9.0 mg, 0.060 mmol), S-Phos (1.9 mg, 4.6x10^{-3} mmol), Na_2CO_3 (6.5 mg, 0.060 mmol). Reaction time 16 h; mp: 242-243 ℃; R_f: 0.33 (AcOEt/Hexane, 1:3); 93% yield as dark green crystals; IR (KBr, cm^{-1}): 3135 (w), 3059 (w), 3026 (w), 1719 (s), 1603 (m), 1545 (s), 1480 (s), 1411 (s), 1353 (w), 1081 (s), 994 (s), 959 (s), 905 (w), 873 (w), 838 (w), 780 (s), 745 (s), 692 (s), 647 (w), 593 (w), 578 (w), 499 (w); ^{1}H NMR (500 MHz, $CDCl_3$) δ 7.98 (s, 2H), 7.92 (s, 1H), 7.86 (s, 1H), 7.79 (d, *J* = 1.7 Hz, 1H), 7.74 (dd, *J* = 8.5, 1.8 Hz, 1H), 7.61 (t, *J* = 7.6 Hz, 1H), 7.56 – 7.52 (m, 3H), 7.47 (t, *J* = 7.7 Hz, 1H), 7.37 (t, *J* = 7.6 Hz, 2H), 7.29 (t, *J* = 7.5 Hz, 1H), 7.17 (s, 2H), 6.94 (d, *J* = 4.0 Hz, 2H), 6.59 (d, *J* = 3.2 Hz, 2H); ^{13}C NMR (126 MHz, $CDCl_3$) δ 159.9, 155.0, 145.2, 145.0, 139.0, 138.0, 137.2, 135.0, 134.7, 133.2, 131.4, 130.3, 130.0, 129.9, 129.8, 129.1, 128.9, 128.2, 128.0, 127.8, 127.4, 126.9, 126.8, 119.9, 119.2, 117.0. HRMS (ESI+) *m/z* calcd for $C_{32}H_{22}BF_2N_2O_2$ $[M+H]^+$ 515.1742. Found 515.1744.

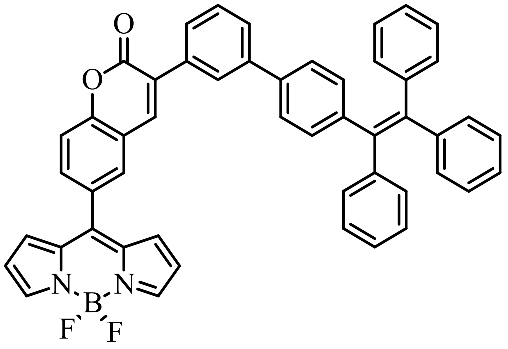

7i

Synthesis of 7i. According to general procedure. **6** (15 mg, 0.030 mmol), 4-(1,2,2-triphenylvinyl)phenylboronic acid (23.0 mg, 0.060 mmol), $Pd(OAc)_2$ (9.0 mg, 0.060 mmol), S-Phos (1.9 mg, 4.6x10^{-3} mmol), Na_2CO_3 (6.5 mg, 0.060 mmol). Reaction time 6.5 h; mp: 179-180 ℃; R_f: 0.32 (AcOEt/Hexane, 1:3); 82% yield as orange solid; IR (KBr, cm^{-1}): 3053 (w), 3025 (w), 1737 (s), 1599 (w), 1555 (s), 1480 (m), 1412 (m), 1387 (s), 1355 (w), 1261 (s), 1199 (m), 1113 (s), 1079 (s), 997 (m), 957 (m), 843 (w), 775 (w), 743 (w), 699 (s), 577 (w); ^1H NMR (500 MHz, $CDCl_3$) δ 7.99 (s, 2H), 7.91 (s, 1H), 7.88 (s, 1H), 7.77 (d, *J* = 1.5 Hz, 1H), 7.74 (dd, *J* = 8.5, 1.7 Hz, 1H), 7.68 (d, *J* = 7.7 Hz, 1H), 7.63 (d, *J* = 7.8 Hz, 1H), 7.54 (d, *J* = 8.5 Hz, 1H), 7.50 (t, *J* = 7.7 Hz, 1H), 7.39 (d, *J* = 8.2 Hz, 2H), 7.13 – 7.03 (m, 17H), 6.93 (d, *J* = 3.9 Hz, 2H), 6.59 (d, *J* = 2.9 Hz, 2H); ^{13}C NMR (126 MHz, $CDCl_3$) δ 159.9, 155.0, 145.2, 145.0, 143.8, 143.4, 141.5, 141.4, 140.5, 139.0, 138.4, 135.0, 134.7, 133.2, 132.0, 131.5, 131.5, 131.5, 131.4, 130.3, 130.1, 129.9, 129.1, 128.1, 128.0, 127.9, 127.8, 127.5, 127.2, 126.7, 126.7, 126.6, 126.5, 119.9, 119.2, 117.0. HRMS (ESI+) *m/z* calcd for $C_{50}H_{33}BF_2N_2O_2$ $[M+H]^+$ 743.2684. Found 743.2664.

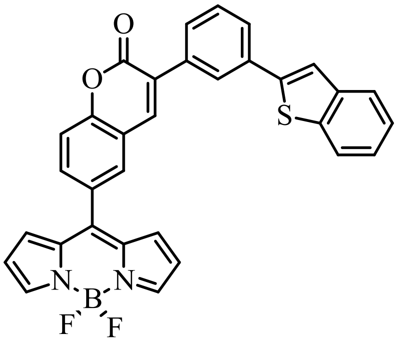

7j

Synthesis of 7j. According to general procedure. **6** (15 mg, 0.030 mmol), benzo[b]thien-2-ylboronic acid (10.9 mg, 0.060 mmol), $Pd(OAc)_2$ (9.0 mg, 0.060 mmol), S-Phos (1.9 mg, 4.6x10^{-3} mmol), Na_2CO_3 (6.5 mg, 0.060 mmol). Reaction time 7 h; mp: 294-296 ℃; R_f: 0.46 (AcOEt/Hexane, 1:3); 77% yield as orange solid; IR (KBr, cm^{-1}): 3055 (C-H_{AR}, w), 1720 (C=O, s), 1601 (w), 1545 (s), 1480 (m), 1411 (s), 1386 (s), 1352 (w), 1262 (C-O, s), 1202 (s), 1116 (s), 1080 (C-O, s), 993 (w), 948 (w), 906 (w), 828 (w), 780 (w), 746 (w), 727 (w), 687 (w), 647 (w), 583 (w); ^1H NMR (500 MHz, $CDCl_3$) δ. HRMS (ESI+) *m/z* calcd for $C_{32}H_{20}BF_2N_2O_2S$ $[M+H]^+$ 545.1307. Found 545.1315.

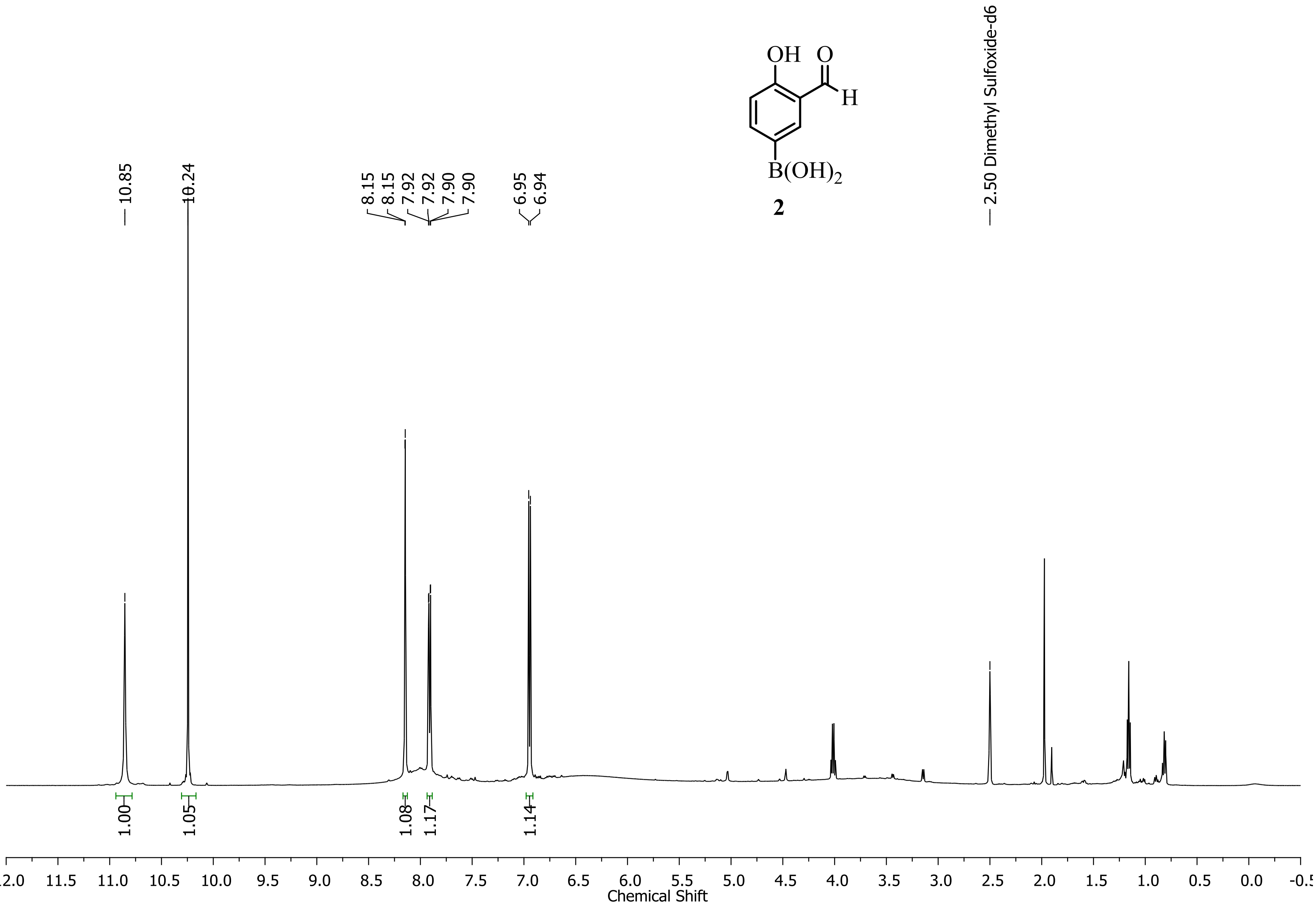
OH
O
H
B(OH)2
2
10.85
10.24
8.15
8.15
7.92
7.92
7.90
7.90
6.95
6.94
2.50 Dimethyl Sulfoxide-d6
1.00
1.05
1.08
1.17
1.14
12.0 11.5 11.0 10.5 10.0 9.5 9.0 8.5 8.0 7.5 7.0 6.5 6.0 5.5 5.0 4.5 4.0 3.5 3.0 2.5 2.0 1.5 1.0 0.5 0.0 -0.5
Chemical Shift

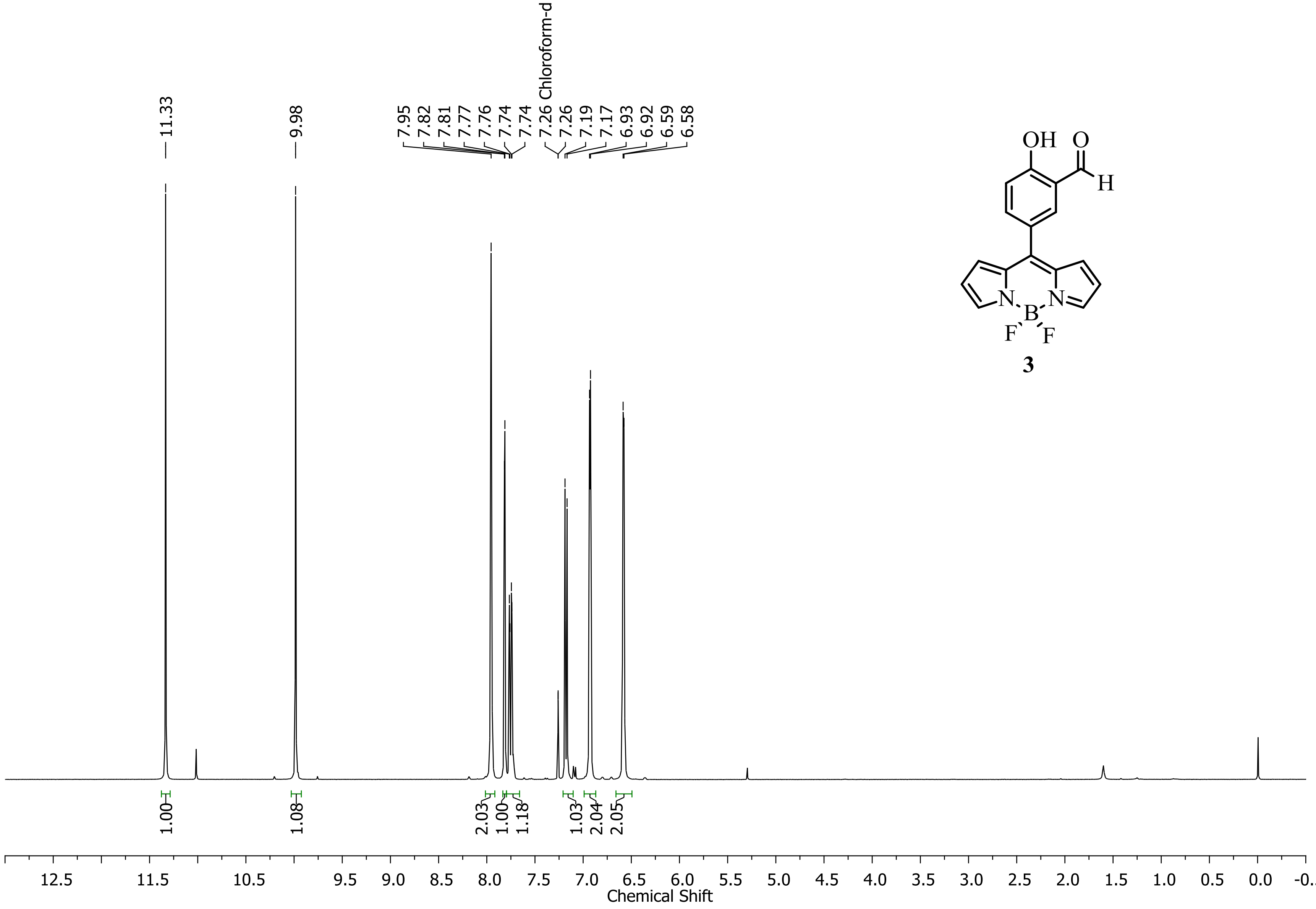

11.33
9.98
7.95
7.82
7.81
7.77
7.76
7.74
7.74
7.26 Chloroform-d
7.26
7.19
7.17
6.93
6.92
6.59
6.58
1.00
1.08
2.03
1.00
1.18
1.03
2.04
2.05
12.5
11.5
10.5
9.5
9.0
8.5
8.0
7.5
7.0
6.5
6.0
5.5
5.0
4.5
4.0
3.5
3.0
2.5
2.0
1.5
1.0
0.5
0.0
Chemical Shift
OH
O
H
N
B
N
F
F

3

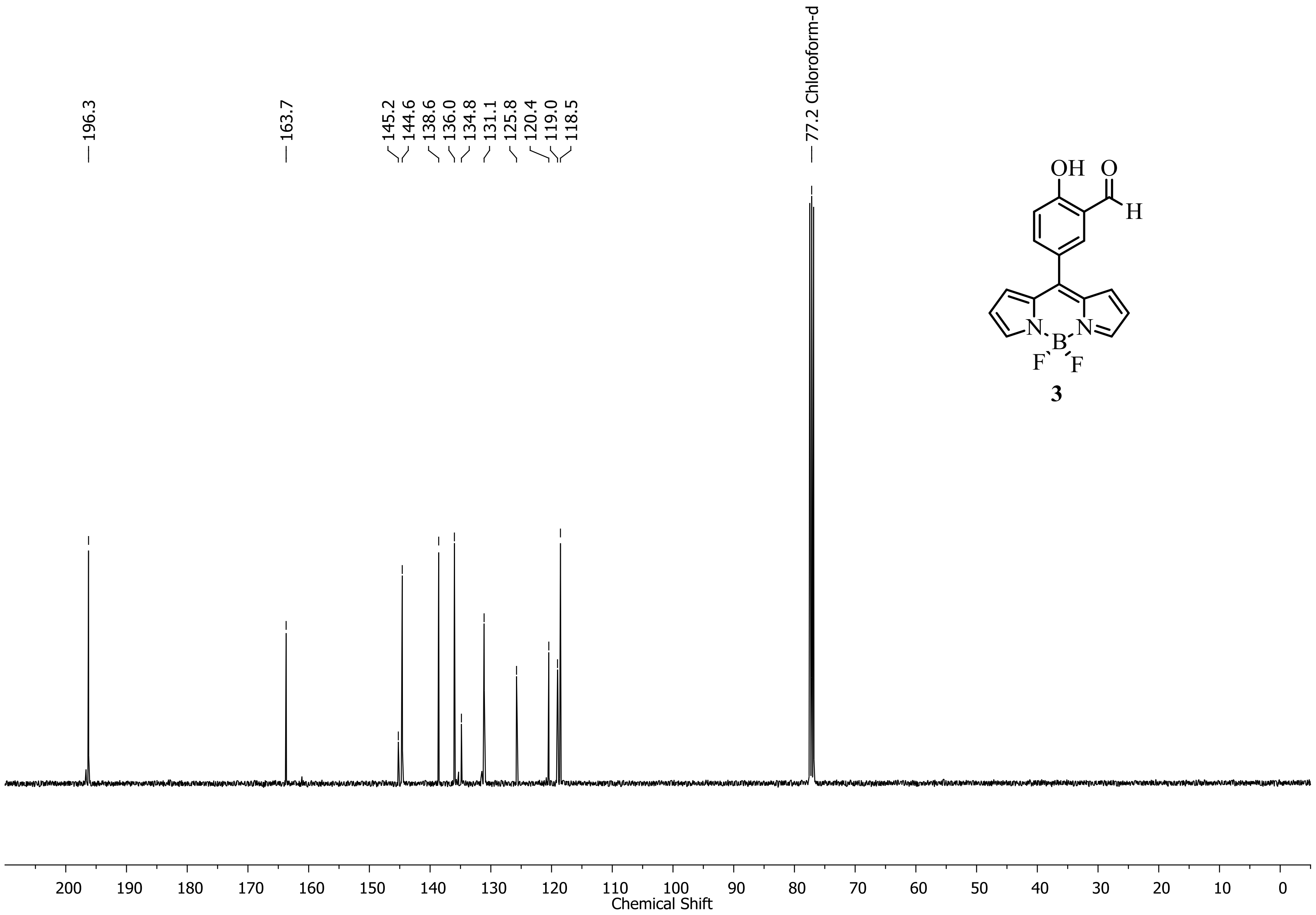
196.3
163.7
145.2
144.6
138.6
136.0
134.8
131.1
125.8
120.4
119.0
118.5
77.2 Chloroform-d
OH
O
H
N
B
N
F
F
3
200
190
180
170
160
150
140
130
120
110
100
90
80
70
60
50
40
30
20
10
0
Chemical Shift

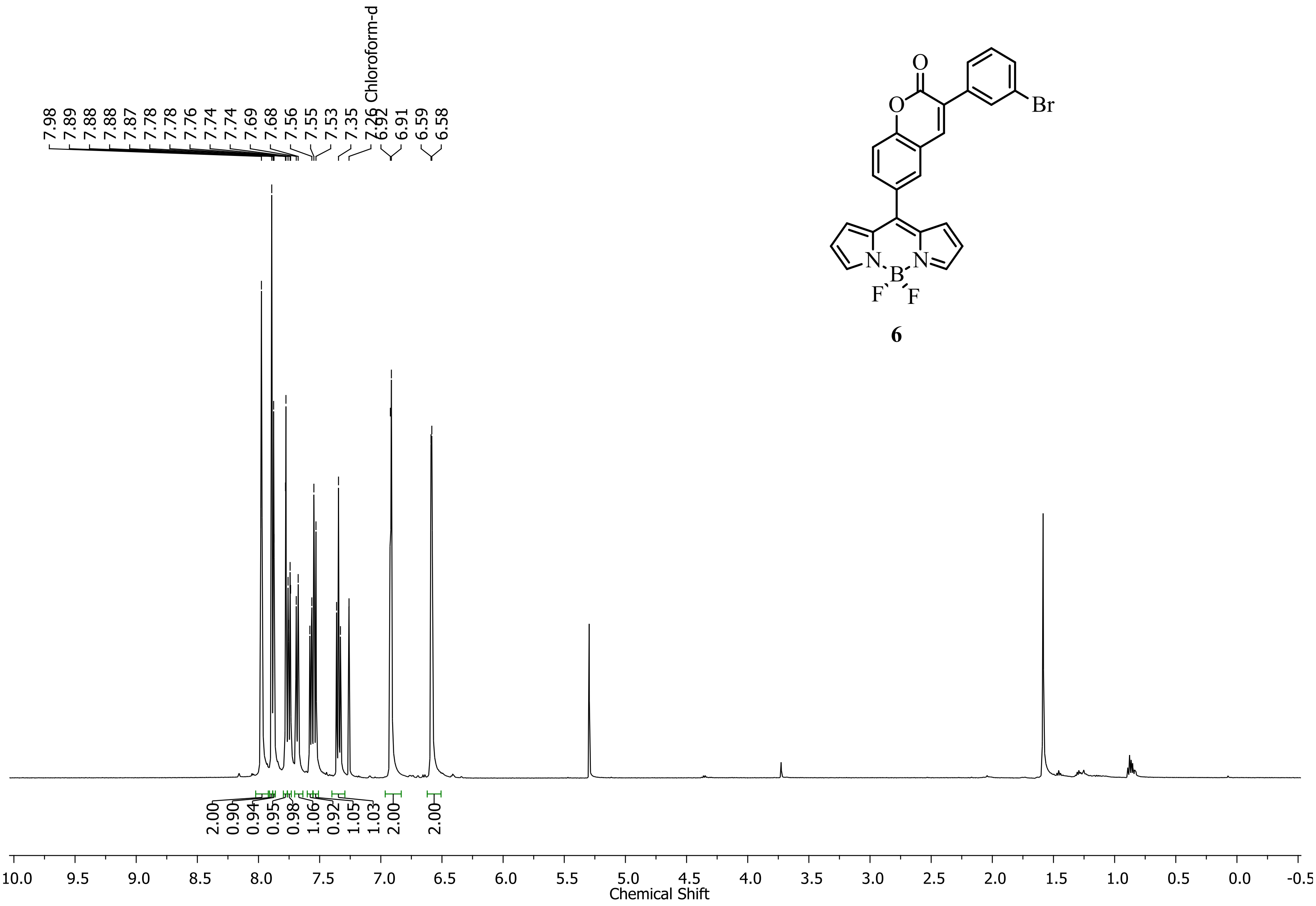

7.98
7.89
7.88
7.88
7.87
7.78
7.78
7.76
7.74
7.74
7.69
7.68
7.56
7.55
7.53
7.35
7.26 Chloroform-d
6.92
6.91
6.59
6.58
2.00
0.90
0.94
0.95
0.98
1.06
0.92
1.05
1.03
2.00
2.00
10.0
9.5
9.0
8.5
8.0
7.5
7.0
6.5
6.0
5.5
5.0
4.5
4.0
3.5
3.0
2.5
2.0
1.5
1.0
0.5
0.0
-0.5
Chemical Shift
O
O
Br
N
B
N
F
F

6

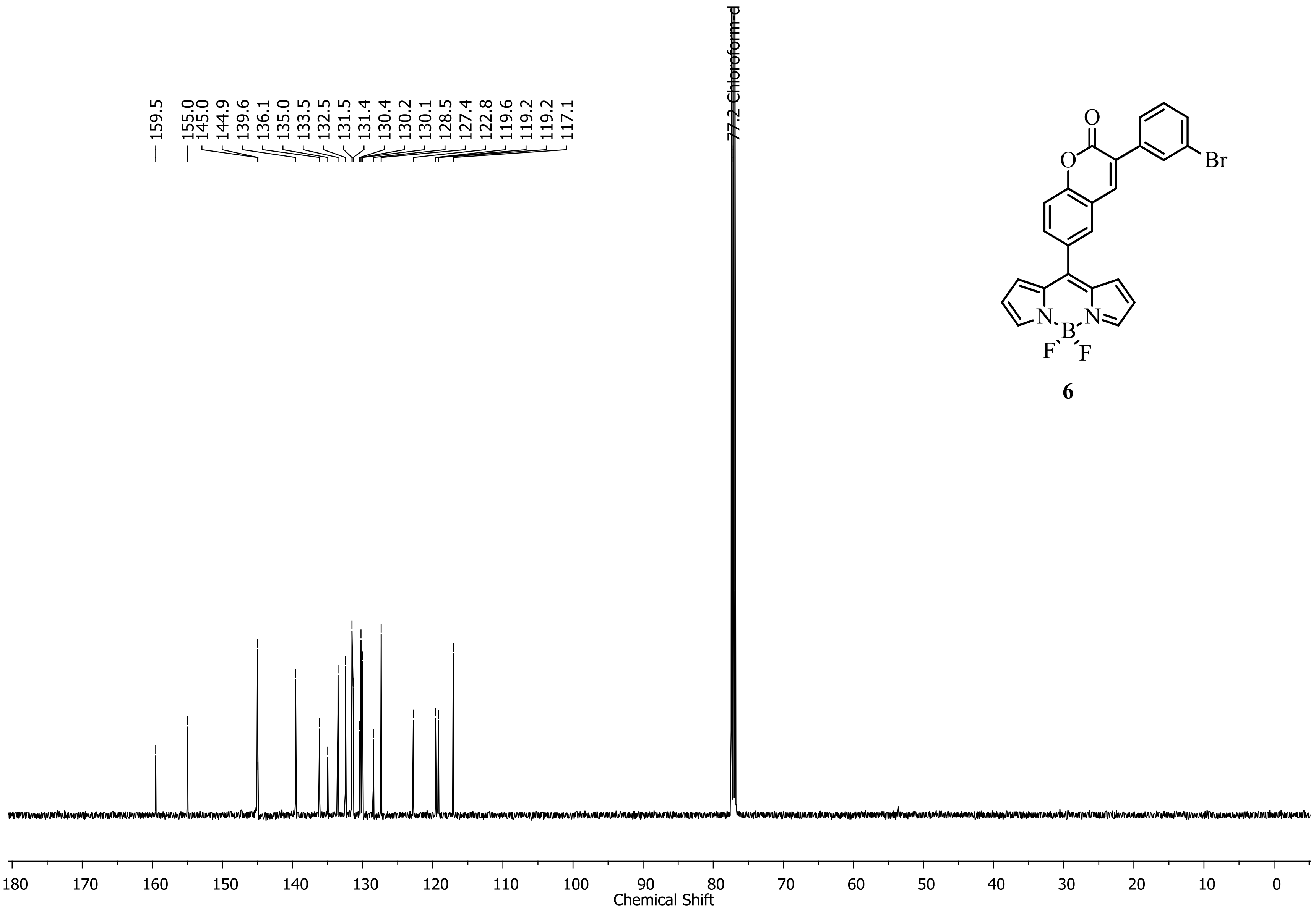
159.5
155.0
145.0
144.9
139.6
136.1
135.0
133.5
132.5
131.5
131.4
130.4
130.2
130.1
128.5
127.4
122.8
119.6
119.2
119.2
117.1
77.2 Chloroform-d
O
O
Br
N
B
N
F
F
180 170 160 150 140 130 120 110 100 90 80 70 60 50 40 30 20 10 0
Chemical Shift

6

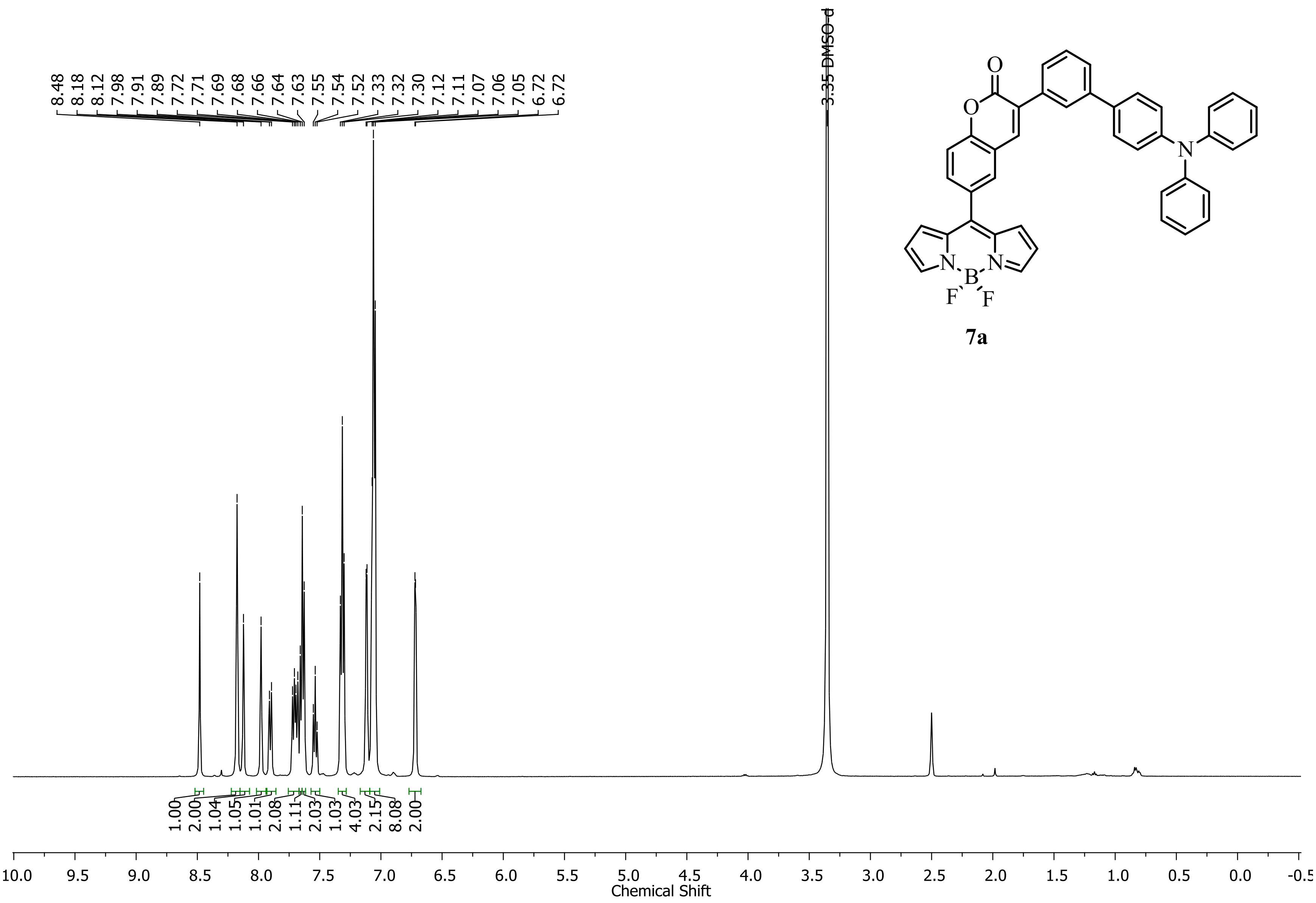

8.48
8.18
8.12
7.98
7.91
7.89
7.72
7.71
7.69
7.68
7.66
7.64
7.63
7.55
7.54
7.52
7.33
7.32
7.30
7.12
7.11
7.07
7.06
7.05
6.72
6.72
3.35 DMSO-d
7a
1.00
2.00
1.04
1.05
1.01
2.08
1.11
2.03
1.03
4.03
2.15
8.08
2.00
10.0
9.5
9.0
8.5
8.0
7.5
7.0
6.5
6.0
5.5
5.0
4.5
4.0
3.5
3.0
2.5
2.0
1.5
1.0
0.5
0.0
Chemical Shift

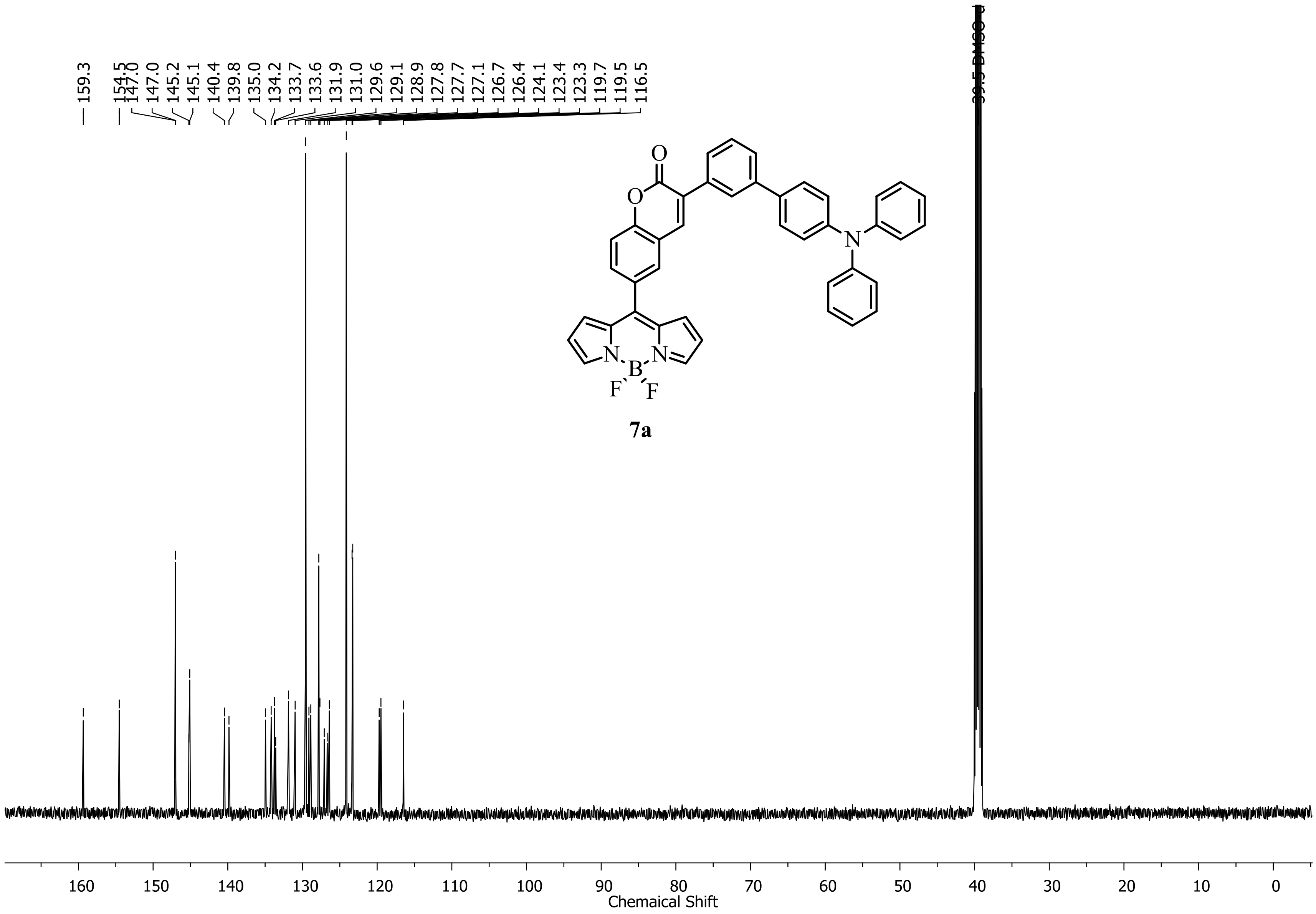
159.3
154.5
147.0
147.0
145.2
145.1
140.4
139.8
135.0
134.2
133.7
133.6
131.9
131.0
129.6
129.1
128.9
127.8
127.7
127.1
126.7
126.4
124.1
123.4
123.3
119.7
119.5
116.5
O
O
N
N
B
N
F
F
7a
160
150
140
130
120
110
100
90
80
70
60
50
40
30
20
10
0
Chemaical Shift

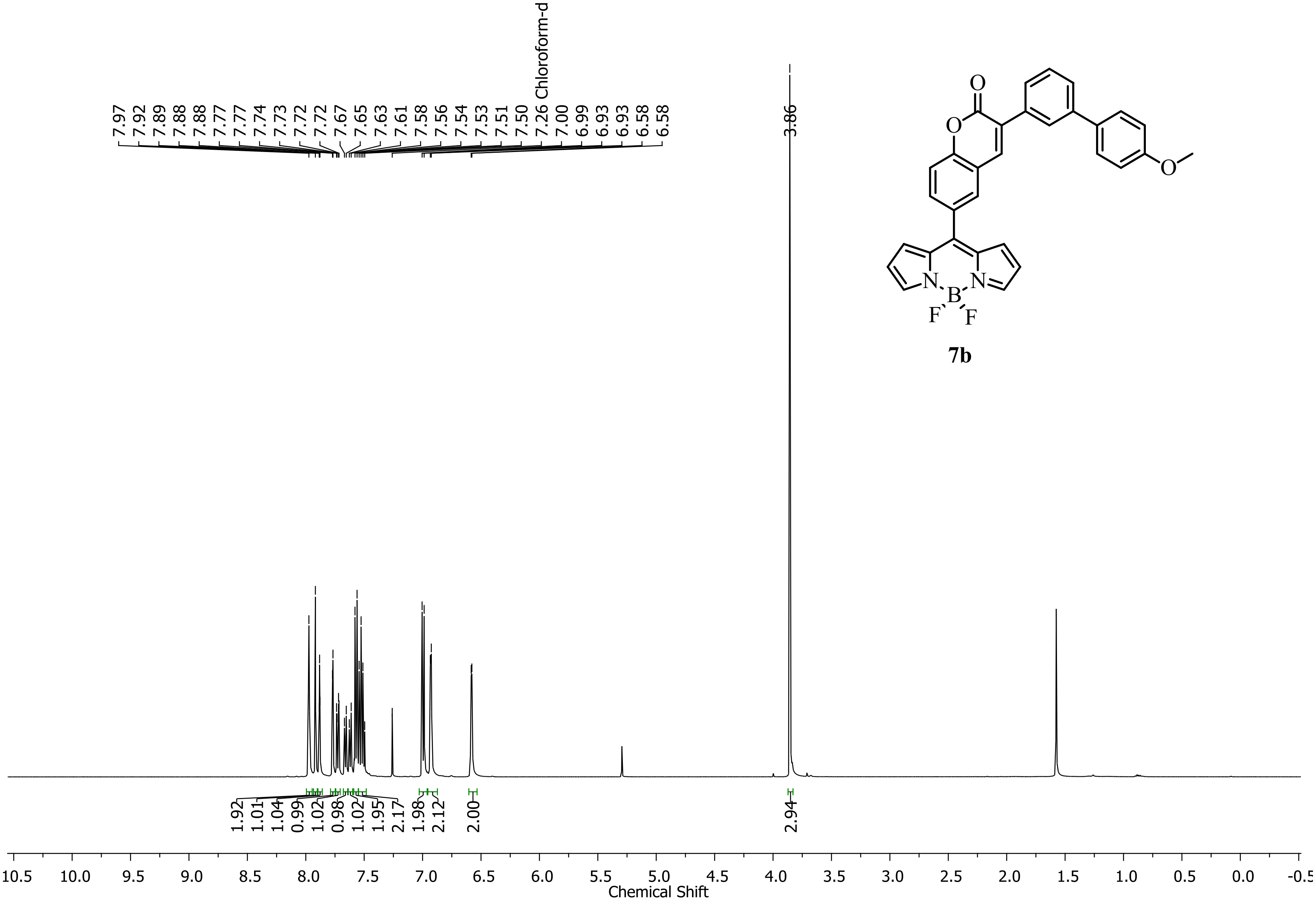
7.97
7.92
7.89
7.88
7.88
7.77
7.77
7.74
7.73
7.72
7.72
7.67
7.65
7.63
7.61
7.58
7.56
7.54
7.53
7.51
7.50
7.26 Chloroform-d
7.00
6.99
6.93
6.93
6.58
6.58
3.86
1.92
1.01
1.04
0.99
1.02
0.98
1.02
1.95
2.17
1.98
2.12
2.00
2.94
10.5 10.0 9.5 9.0 8.5 8.0 7.5 7.0 6.5 6.0 5.5 5.0 4.5 4.0 3.5 3.0 2.5 2.0 1.5 1.0 0.5 0.0 -0.5
Chemical Shift
O
O
O
N
B
N
F
F

7b

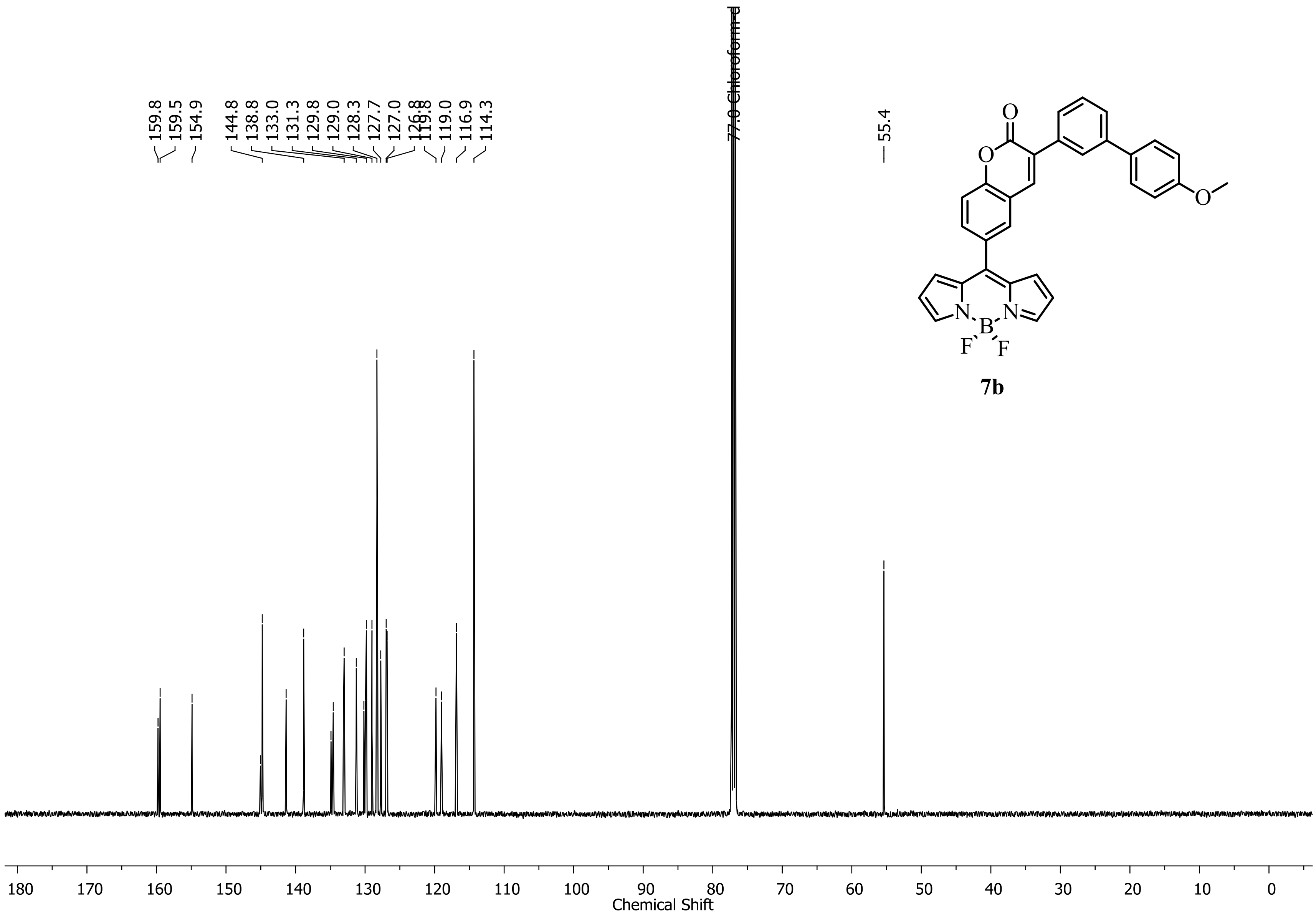
159.8
159.5
154.9
144.8
138.8
133.0
131.3
129.8
129.0
128.3
127.7
127.0
126.8
119.8
119.0
116.9
114.3
77.0 Chloroform-d
55.4
7b
180
170
160
150
140
130
120
110
100
90
80
70
60
50
40
30
20
10
0
Chemical Shift

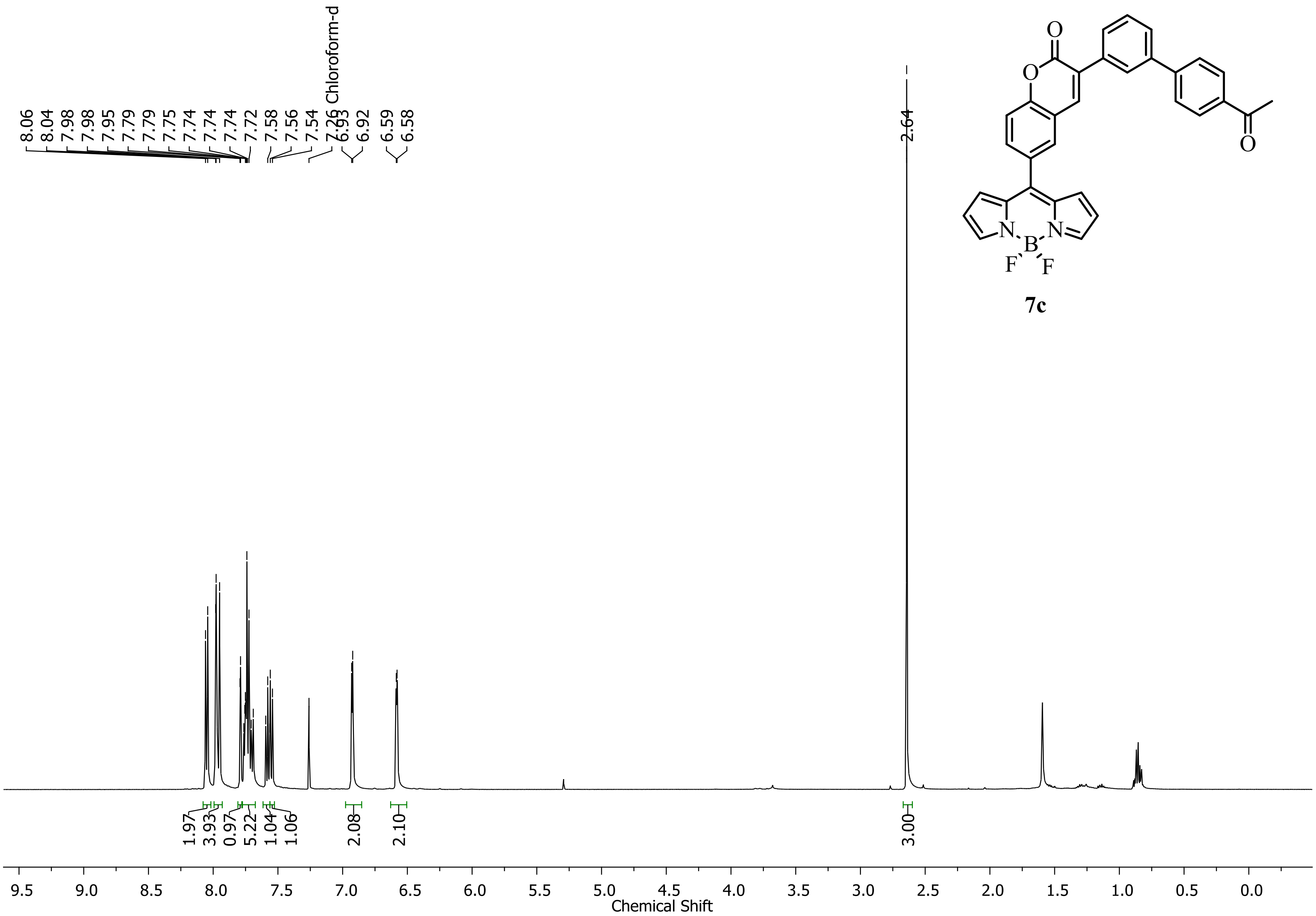

8.06
8.04
7.98
7.98
7.95
7.79
7.79
7.75
7.74
7.74
7.74
7.72
7.58
7.56
7.54
7.26 Chloroform-d
6.93
6.92
6.59
6.58
2.64
1.97
3.93
0.97
5.22
1.04
1.06
2.08
2.10
3.00
9.5 9.0 8.5 8.0 7.5 7.0 6.5 6.0 5.5 5.0 4.5 4.0 3.5 3.0 2.5 2.0 1.5 1.0 0.5 0.0
Chemical Shift
O
O
N
B
N
F
F
O
7c

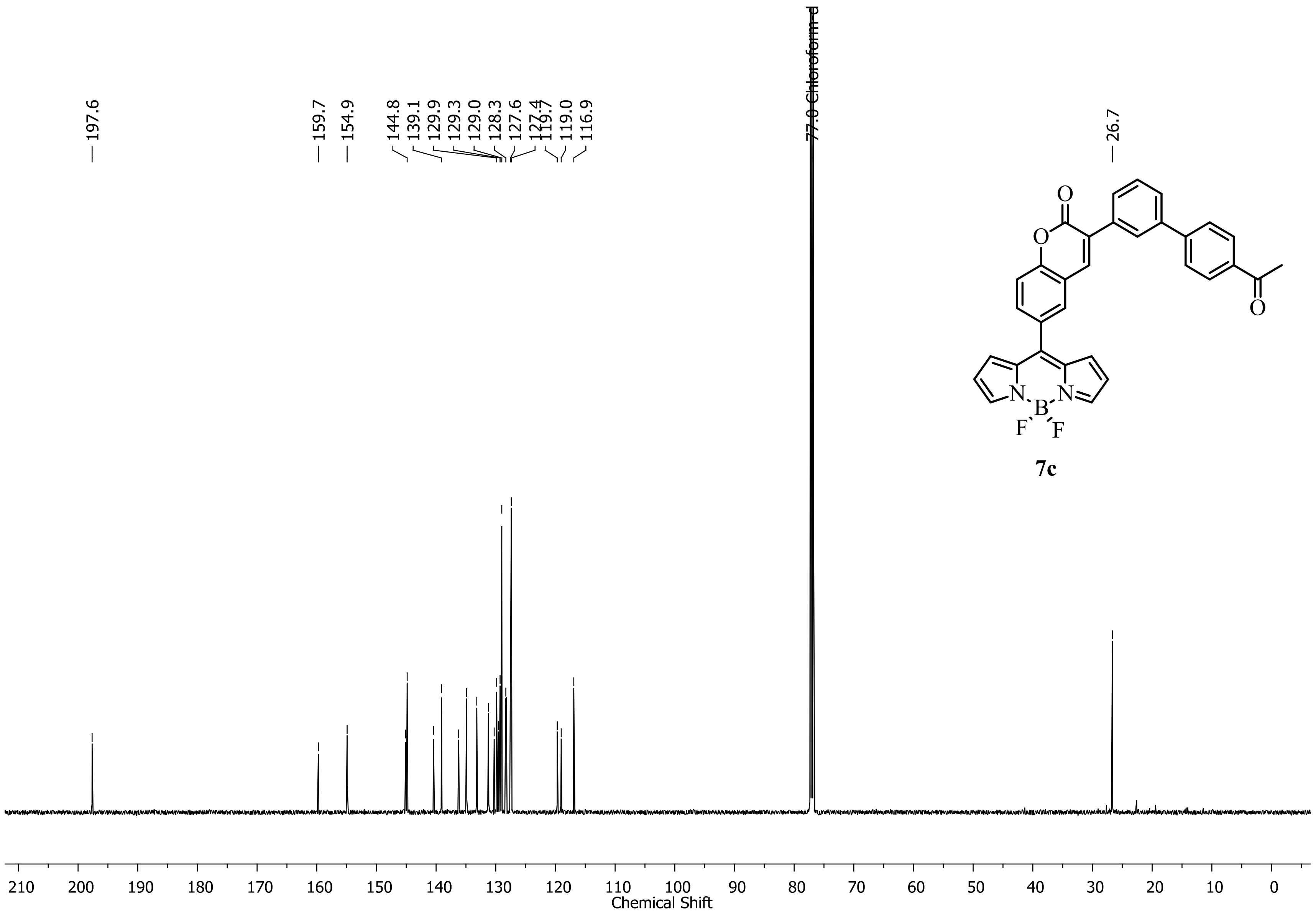
197.6
159.7
154.9
144.8
139.1
129.9
129.3
129.0
128.3
127.6
127.4
119.7
119.0
116.9
77.0 Chloroform-d
26.7
O
O
O
N
B
N
F
F
7c
210 200 190 180 170 160 150 140 130 120 110 100 90 80 70 60 50 40 30 20 10 0
Chemical Shift

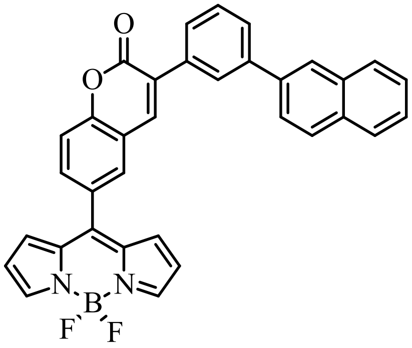

7d

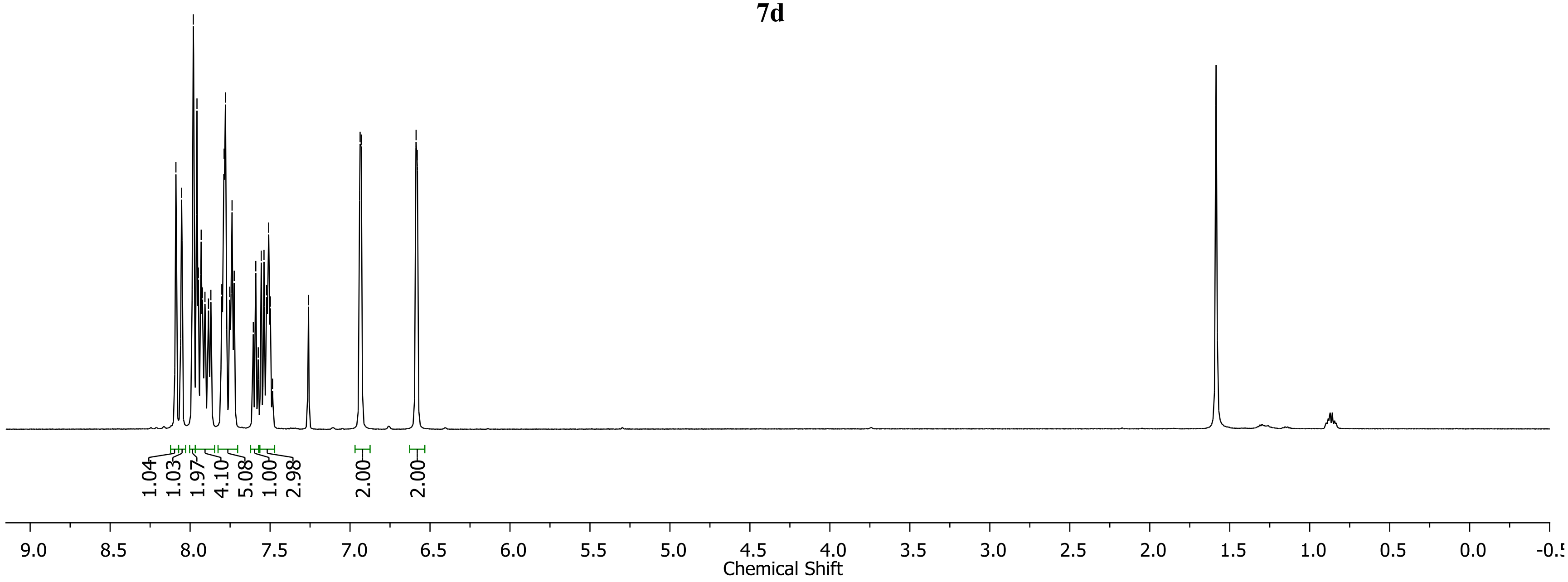
8.09
8.05
7.98
7.96
7.95
7.93
7.79
7.78
7.74
7.59
7.56
7.54
7.51
6.94
6.93
6.59
6.58
1.04
1.03
1.97
4.10
5.08
1.00
2.98
2.00
2.00
Chemical Shift

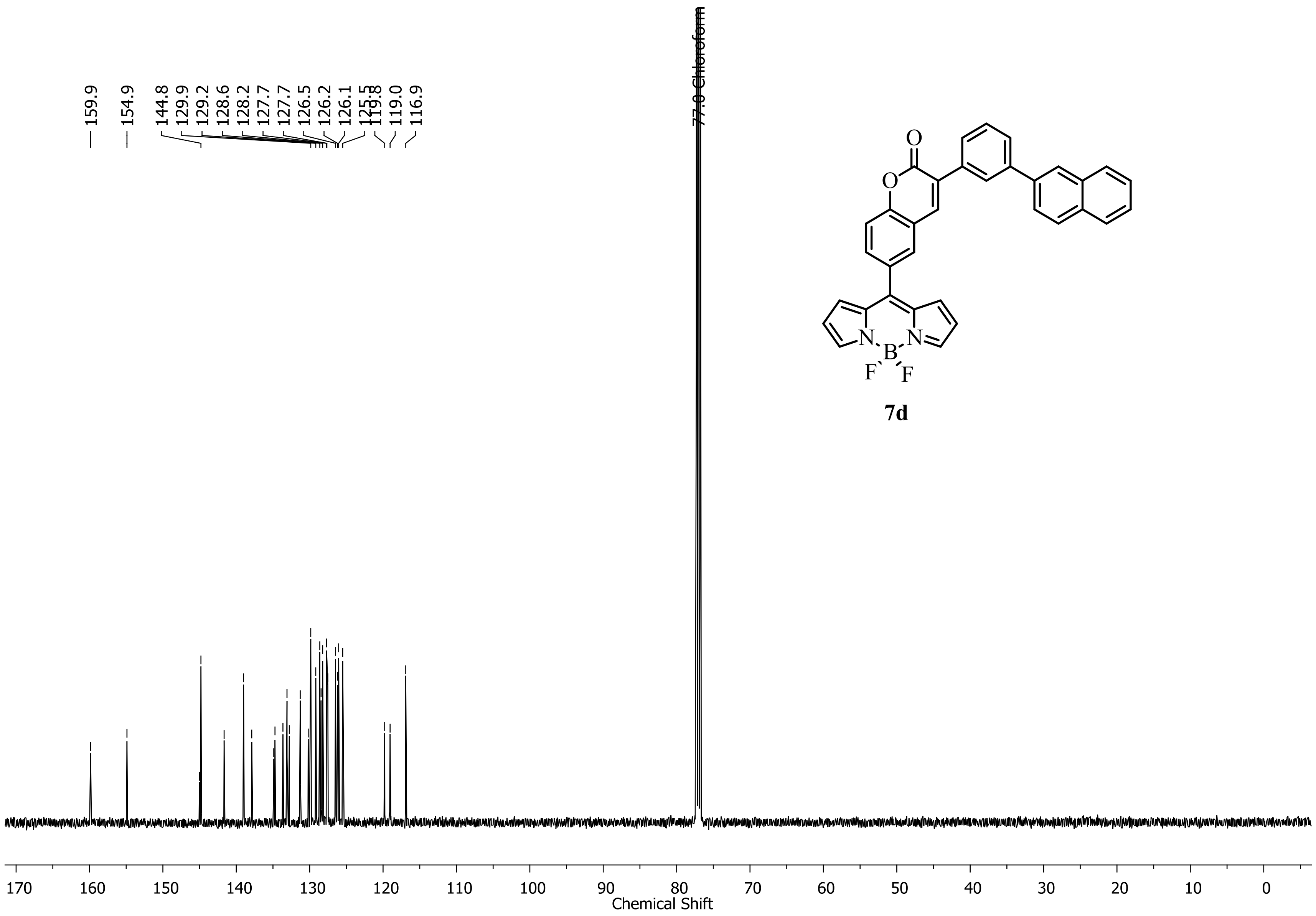
159.9
154.9
144.8
129.9
129.2
128.6
128.2
127.7
127.7
126.5
126.2
126.1
125.5
119.8
119.0
116.9
77.0 Chloroform
O
O
N
B
N
F
F
7d
170
160
150
140
130
120
110
100
90
80
70
60
50
40
30
20
10
0
Chemical Shift

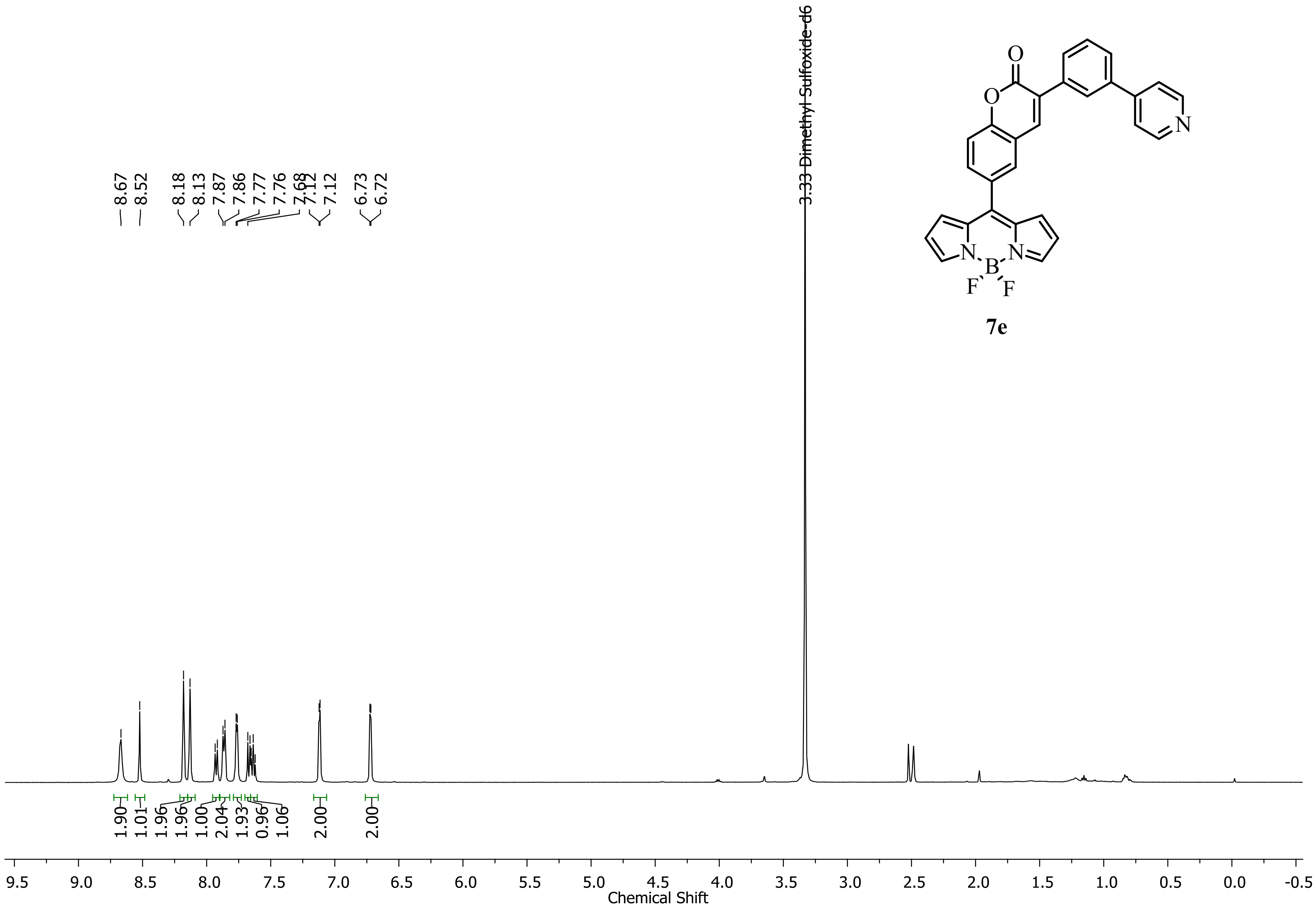
8.67
8.52
8.18
8.13
7.87
7.86
7.77
7.76
7.68
7.12
7.12
6.73
6.72
3.33 Dimethyl Sulfoxide-d6
1.90
1.01
1.96
1.96
1.00
2.04
1.93
0.96
1.06
2.00
2.00
9.5 9.0 8.5 8.0 7.5 7.0 6.5 6.0 5.5 5.0 4.5 4.0 3.5 3.0 2.5 2.0 1.5 1.0 0.5 0.0 -0.5
Chemical Shift
O
O
N
N
B
N
F
F
7e

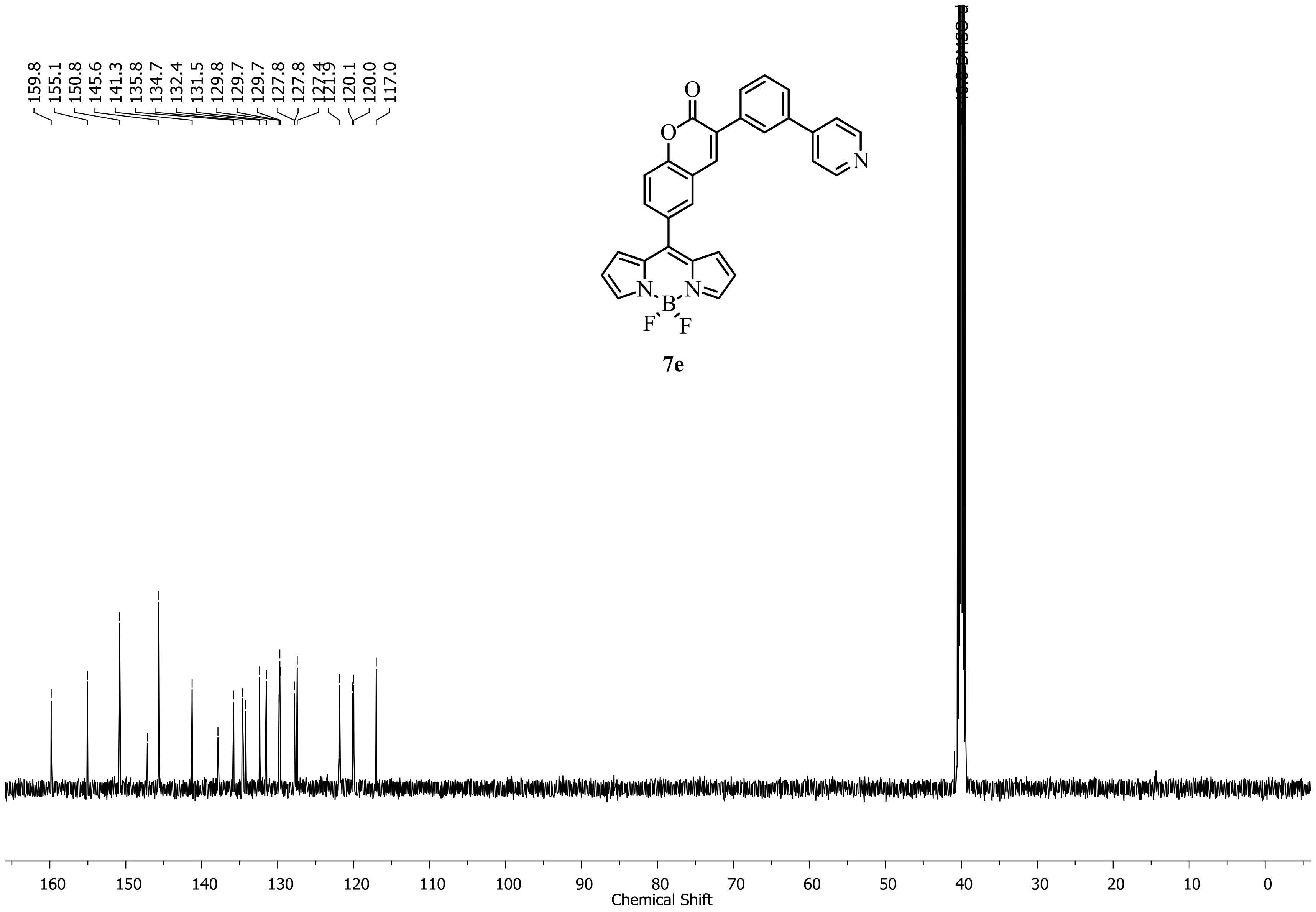
159.8
155.1
150.8
145.6
141.3
135.8
134.7
132.4
131.5
129.8
129.7
129.7
129.7
127.8
127.8
127.4
121.9
120.1
120.0
117.0
O
O
N
N
N
B
F
F
7e
160
150
140
130
120
110
100
90
80
70
60
50
40
30
20
10
0
Chemical Shift

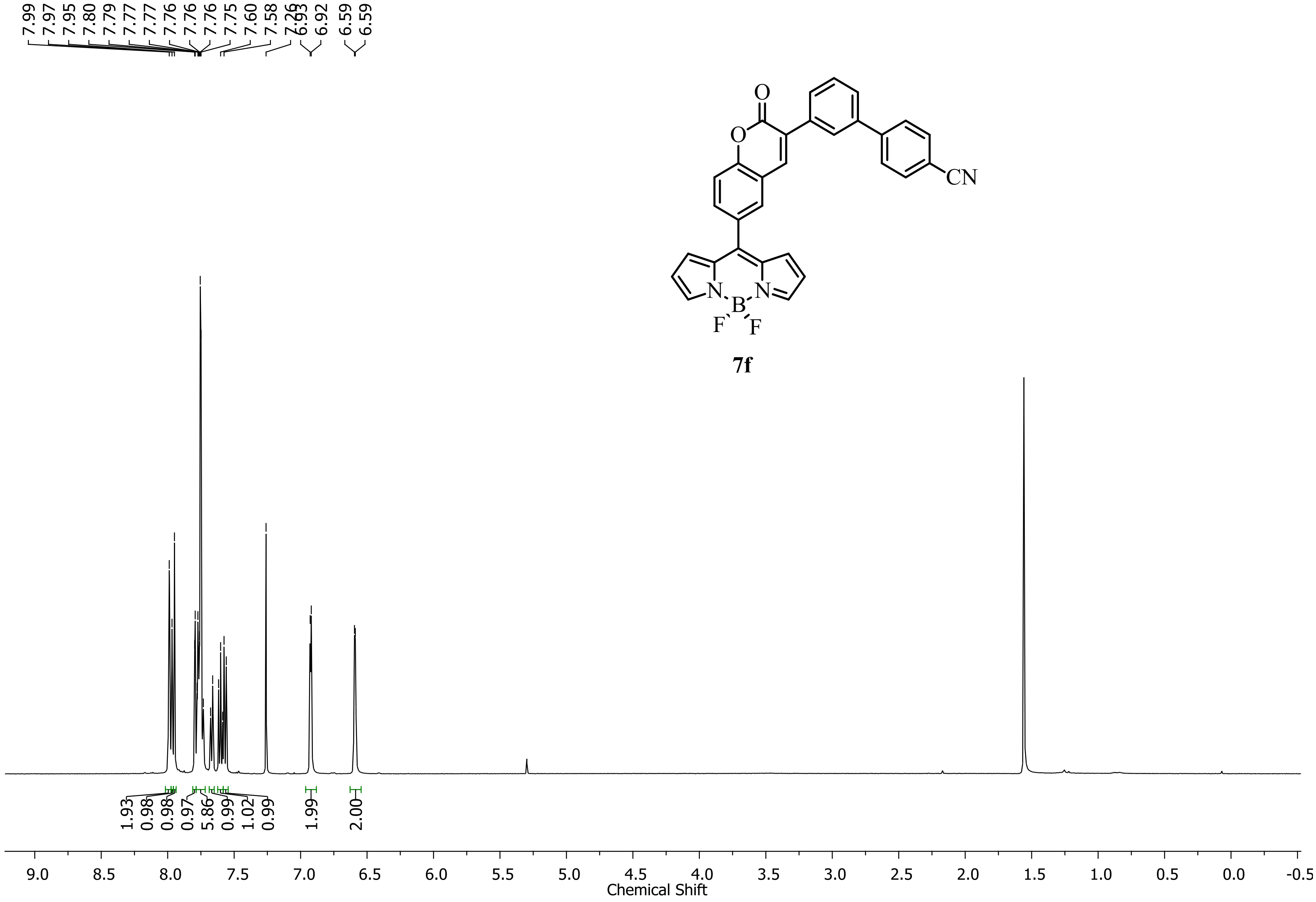
7.99
7.97
7.95
7.80
7.79
7.77
7.77
7.76
7.76
7.76
7.75
7.60
7.58
7.26
6.93
6.92
6.59
6.59
O
O
CN
N
B
N
F
F
7f
1.93
0.98
0.98
0.97
5.86
0.99
1.02
0.99
1.99
2.00
9.0
8.5
8.0
7.5
7.0
6.5
6.0
5.5
5.0
4.5
4.0
3.5
3.0
2.5
2.0
1.5
1.0
0.5
0.0
-0.5
Chemical Shift

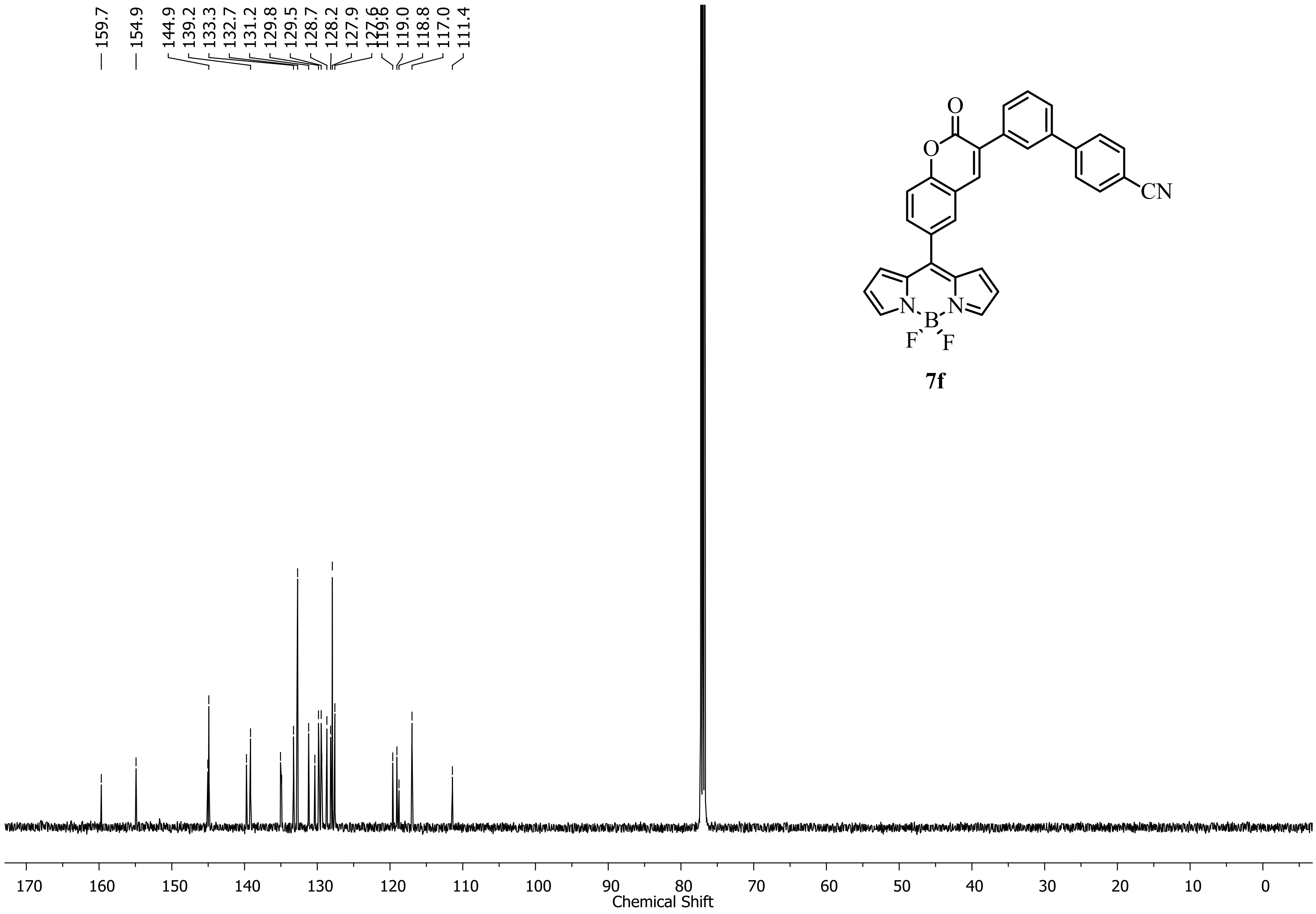
159.7
154.9
144.9
139.2
133.3
132.7
131.2
129.8
129.5
128.7
128.2
127.9
127.6
119.6
119.0
118.8
117.0
111.4
O
O
CN
N
B
N
F
F
7f
170 160 150 140 130 120 110 100 90 80 70 60 50 40 30 20 10 0
Chemical Shift

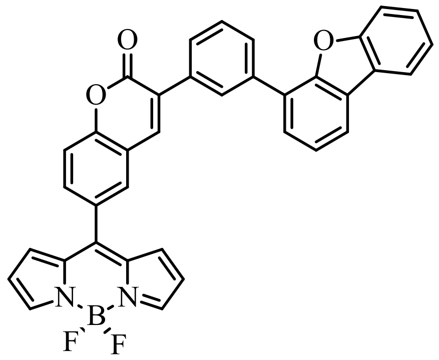

7g

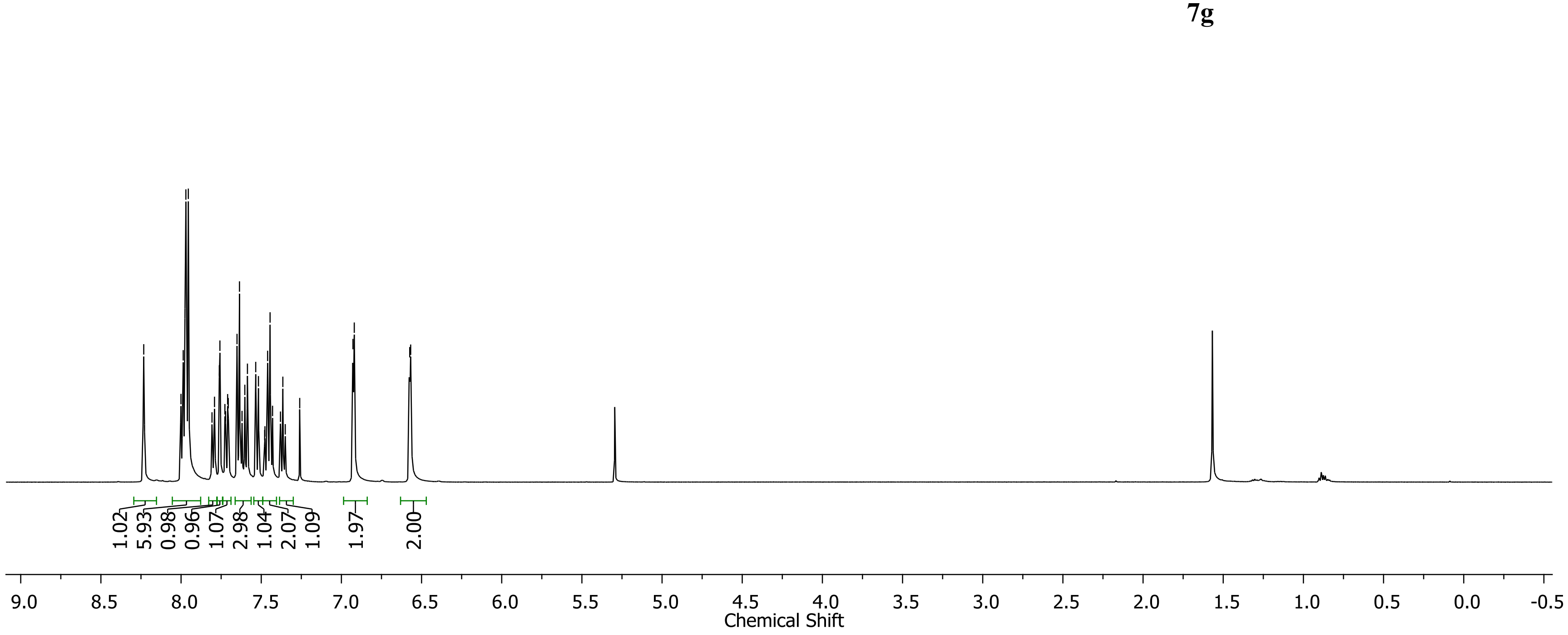
8.23
7.99
7.97
7.95
7.76
7.76
7.65
7.64
7.59
7.53
7.52
7.46
7.44
6.93
6.92
6.57
6.57
1.02
5.93
0.98
0.96
1.07
2.98
1.04
2.07
1.09
1.97
2.00
9.0 8.5 8.0 7.5 7.0 6.5 6.0 5.5 5.0 4.5 4.0 3.5 3.0 2.5 2.0 1.5 1.0 0.5 0.0 -0.5
Chemical Shift

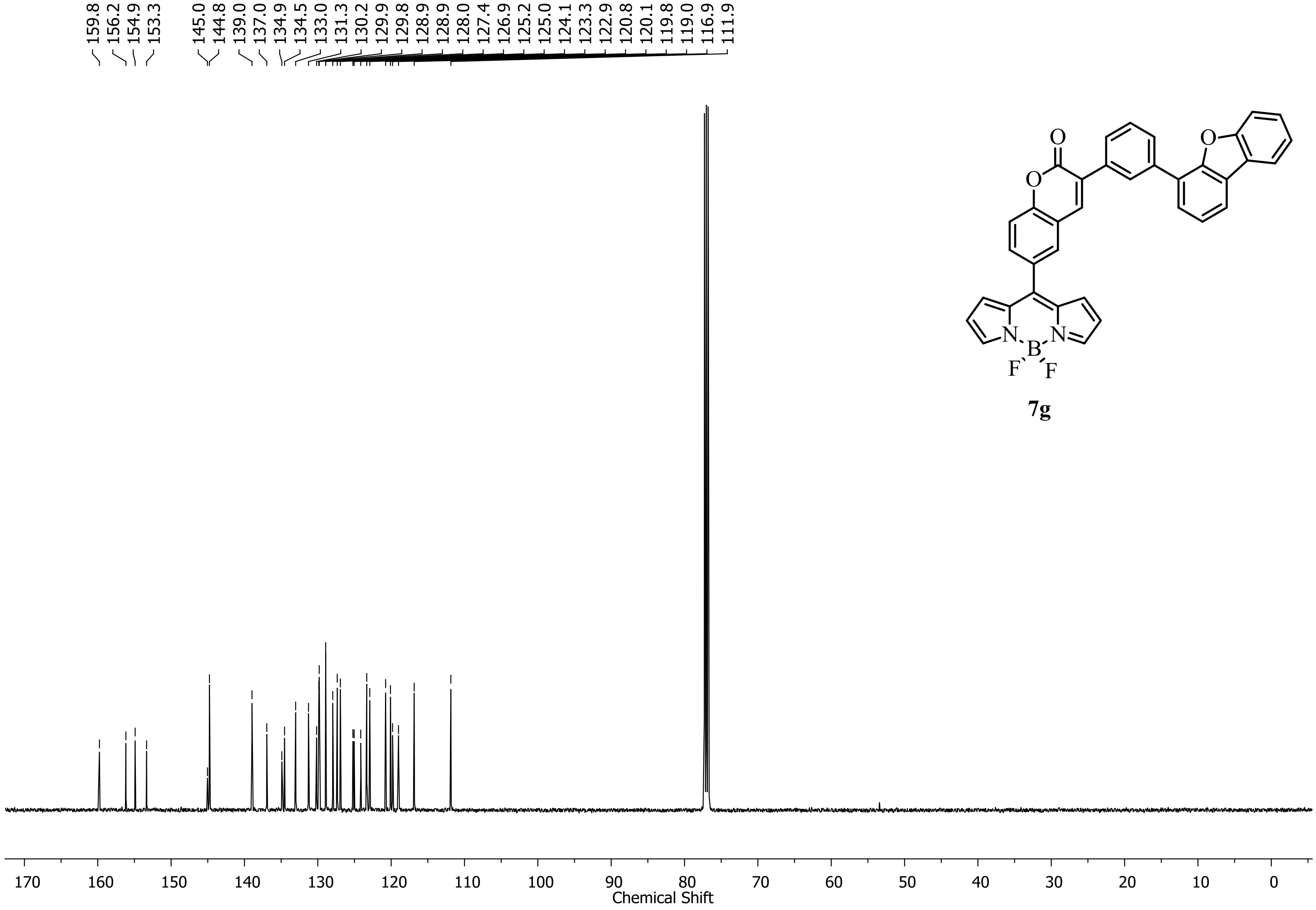

159.8
156.2
154.9
153.3
145.0
144.8
139.0
137.0
134.9
134.5
133.0
131.3
130.2
129.9
129.8
128.9
128.9
128.0
127.4
126.9
125.2
125.0
124.1
123.3
122.9
120.8
120.1
119.8
119.0
116.9
111.9
O
O
O
N
B
N
F
F
7g
170 160 150 140 130 120 110 100 90 80 70 60 50 40 30 20 10 0
Chemical Shift

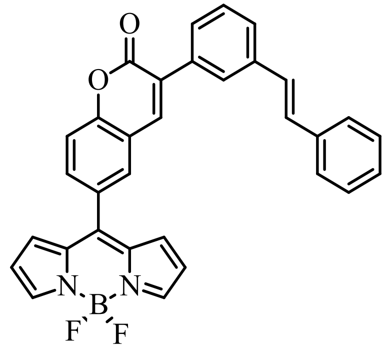

7h

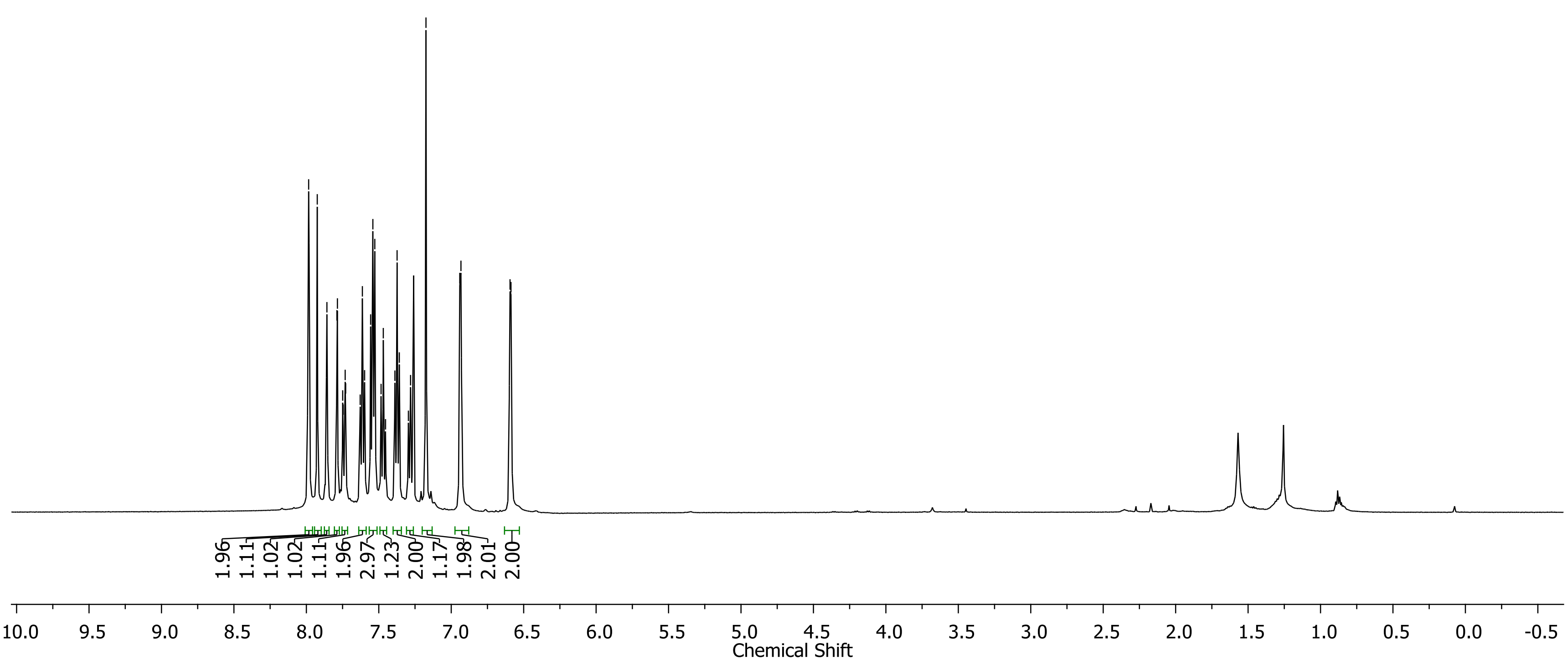
7.98
7.92
7.86
7.79
7.79
7.73
7.61
7.60
7.56
7.54
7.53
7.47
7.39
7.37
7.36
7.28
7.17
6.94
6.93
6.59
6.59
1.96
1.11
1.02
1.02
1.11
1.96
2.97
1.23
2.00
1.17
1.98
2.01
2.00
10.0 9.5 9.0 8.5 8.0 7.5 7.0 6.5 6.0 5.5 5.0 4.5 4.0 3.5 3.0 2.5 2.0 1.5 1.0 0.5 0.0 -0.5
Chemical Shift

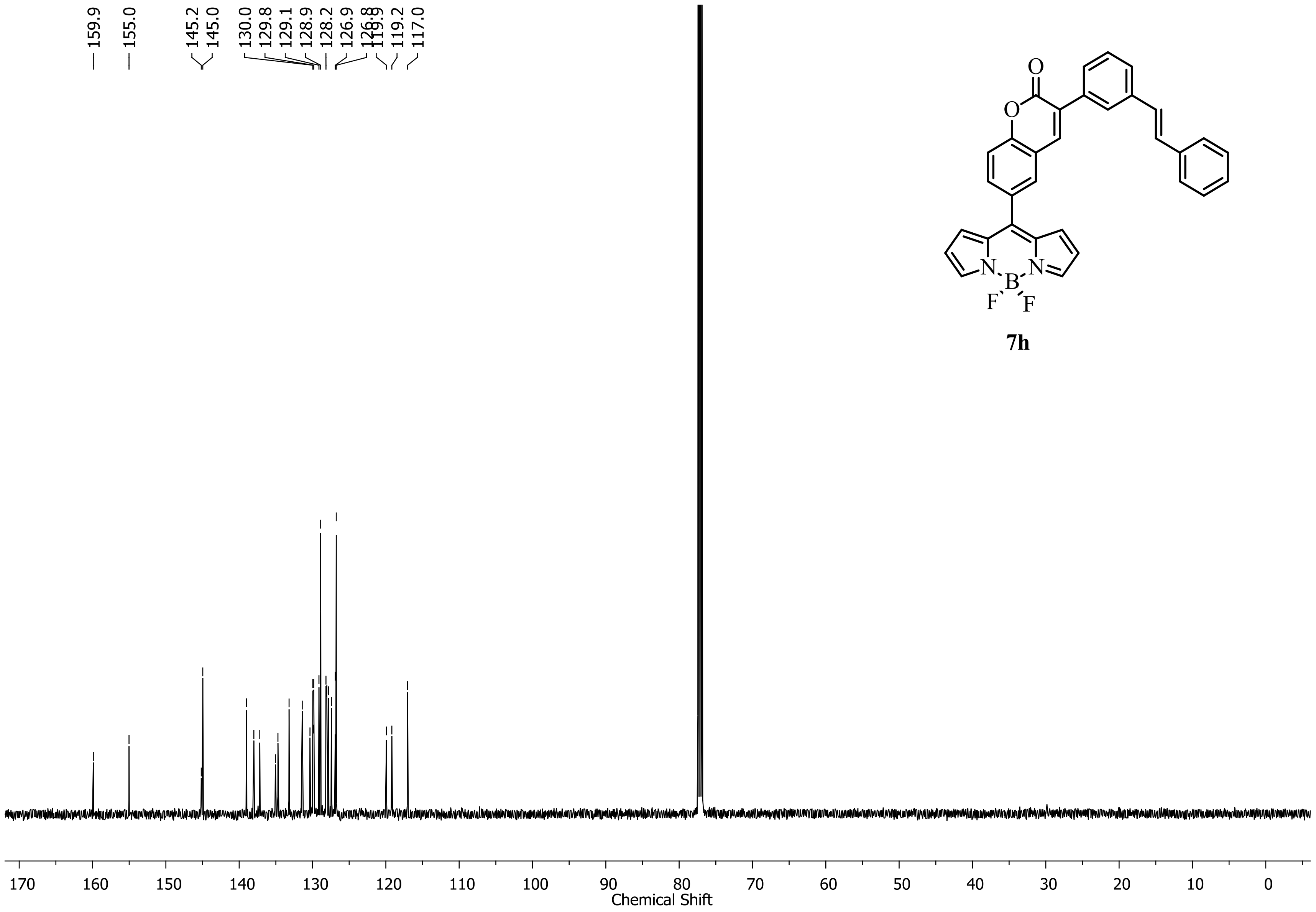
159.9
155.0
145.2
145.0
130.0
129.8
129.1
128.9
128.2
126.9
126.8
119.9
119.2
117.0
170
160
150
140
130
120
110
100
90
80
70
60
50
40
30
20
10
0
Chemical Shift
O
O
N
B
N
F
F

7h

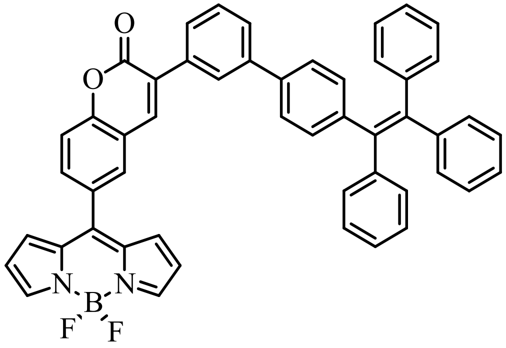
O
O
N
B
N
F
F

7i

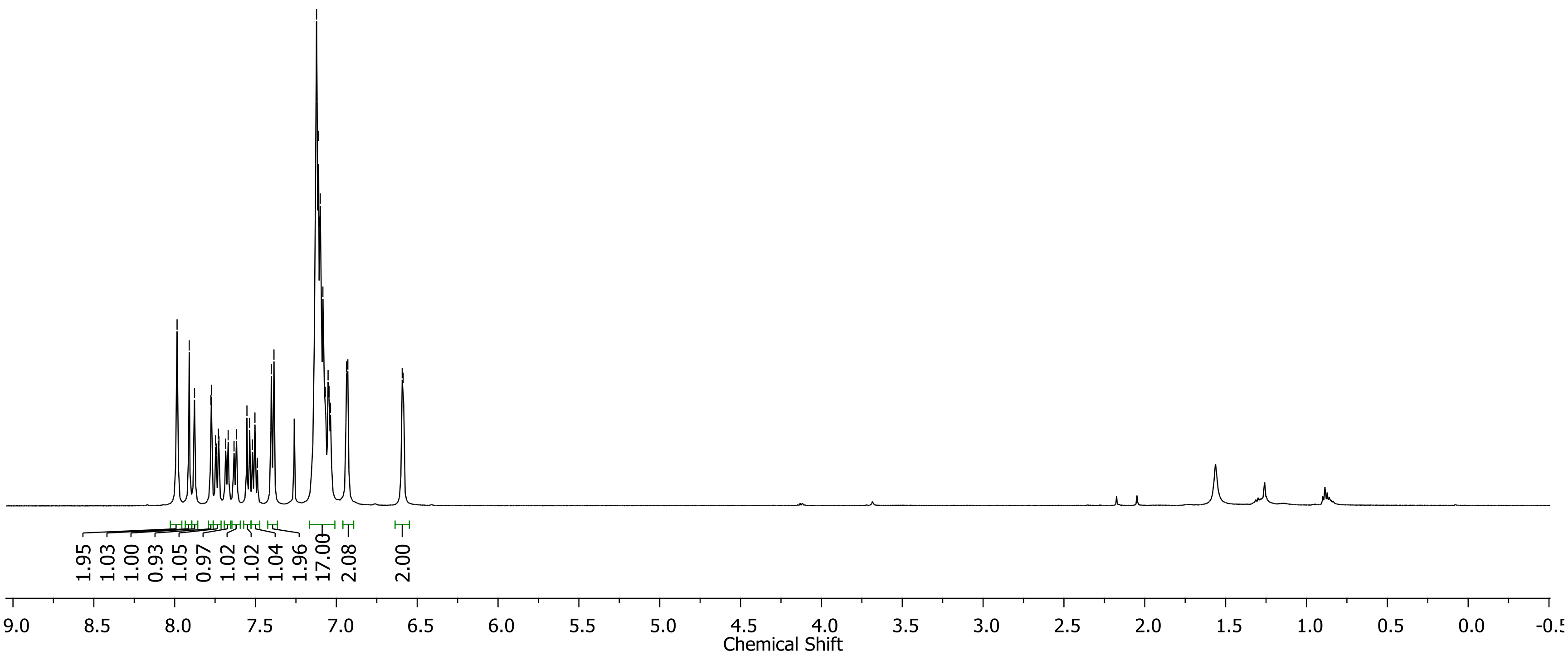
7.99
7.91
7.77
7.40
7.39
7.12
7.11
7.10
7.10
7.08
7.07
7.05
7.05
6.94
6.93
6.59
6.59
1.95
1.03
1.00
0.93
1.05
0.97
1.02
1.02
1.04
1.96
17.00
2.08
2.00
9.0
8.5
8.0
7.5
7.0
6.5
6.0
5.5
5.0
4.5
4.0
3.5
3.0
2.5
2.0
1.5
1.0
0.5
0.0
Chemical Shift

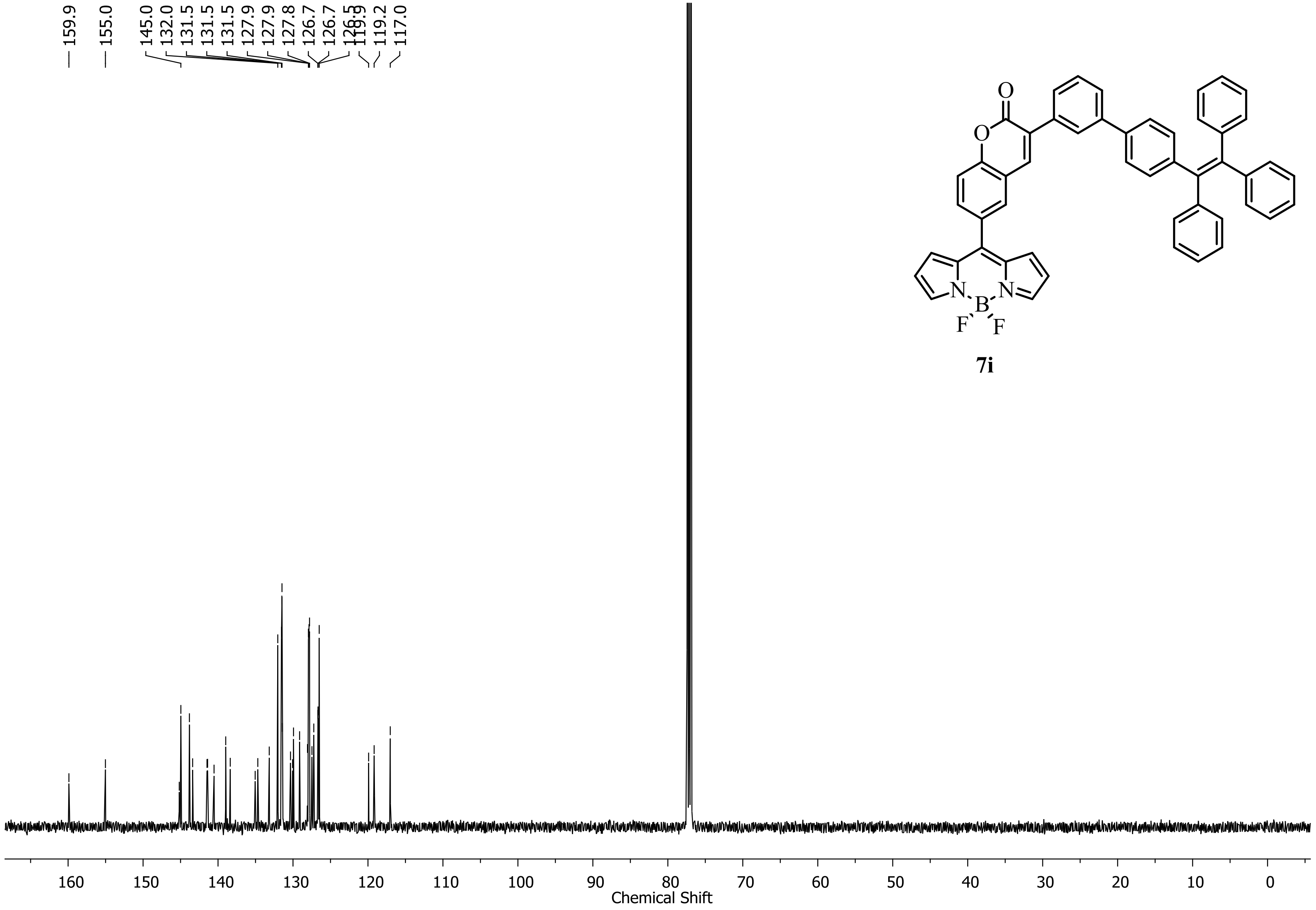
159.9
155.0
145.0
132.0
131.5
131.5
131.5
127.9
127.9
127.8
126.7
126.7
126.5
119.9
119.2
117.0
160 150 140 130 120 110 100 90 80 70 60 50 40 30 20 10 0
Chemical Shift
O
O
N
B
N
F
F

7i

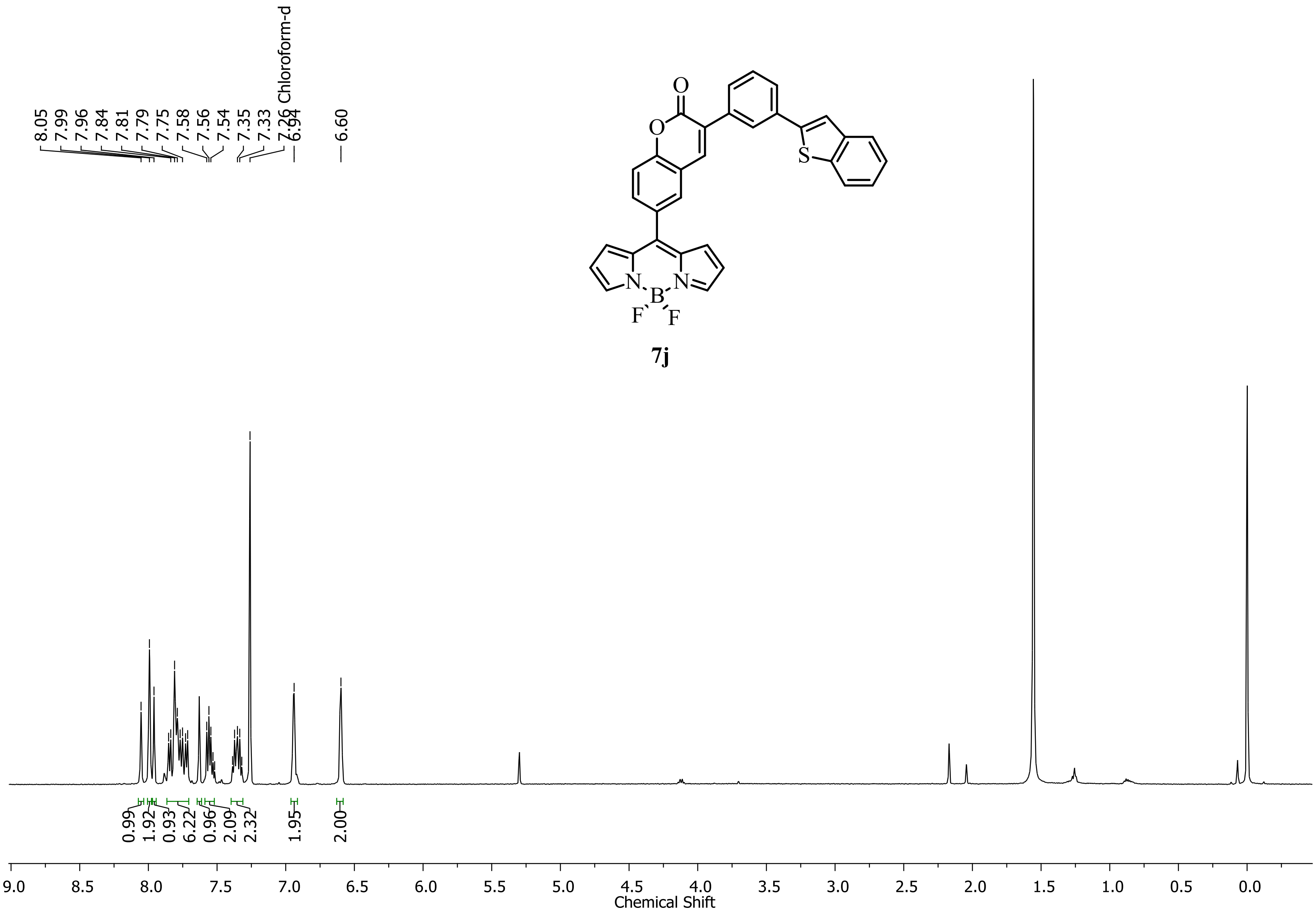
8.05
7.99
7.96
7.84
7.81
7.79
7.75
7.58
7.56
7.54
7.35
7.33
7.26 Chloroform-d
6.94
6.60
7j
0.99
1.92
0.93
6.22
0.96
2.09
2.32
1.95
2.00
Chemical Shift

Table S1. Photophysical properties of the BODIPY bearing 8-coumarin with π-extended delocalized frameworks in diluted solutions of different solvents.

	λ_{ab} (nm)	ε_{max} ($10^4 M^{-1}\cdot cm^{-1}$)	λ_{fl} (nm)	$\Delta\nu_{St}$ (cm^{-1})	ϕ	τ (ps)	k_{fl} ($10^8 s^{-1}$)	k_{nr} ($10^8 s^{-1}$)
**3**								
c-hex	502.5	5.6	517.5	575	0.037	435	0.85	22.1
EtOAc	498.5	5.3	515.0	640	0.032	370	0.86	26.1
AcN	497.0	3.9	514.5	685	0.026	315	0.82	30.9
**6**								
c-hex	504.5	5.7	518.5	535	0.026	290	0.89	33.6
EtOAc	500.0	5.4	517.5	675	0.026	275	0.94	35.4
AcN	499.0	5.0	517.5	715	0.020	235	0.85	41.7
**7a**								
c-hex	504.0	6.1	520.5	630	0.031	330	0.94	29.3
EtOAc	500.0	5.3	517.5	675	0.020	215	0.93	45.6
AcN	499.0	5.1	516.5	680	0.014	175	0.80	56.3
**7b**								
c-hex	504.0	6.1	520.5	630	0.030	330	0.91	29.4
EtOAc	500.0	5.3	517.0	655	0.027	295	0.91	33.3
AcN	499.0	5.1	517.5	715	0.020	235	0.85	41.7
**7c**								
c-hex	504.0	5.8	519.5	590	0.028	310	0.90	31.3
EtOAc	500.0	5.3	517.5	675	0.027	285	0.95	34.1
AcN	499.0	5.0	517.0	700	0.019	235	0.81	41.7
**7d**								
c-hex	504.0	6.5	520.0	610	0.030	330	0.91	29.4
EtOAc	500.0	5.6	518.0	695	0.028	265	1.05	36.6
AcN	499.0	5.2	518.0	735	0.020	235	0.85	41.7
**7e**								
c-hex	504.0	4.5	520.5	630	0.023	295	0.80	33.1
EtOAc	500.0	4.6	517.5	675	0.021	285	0.74	34.3
AcN	499.0	4.7	518.0	735	0.017	240	0.71	40.9
**7f***								
EtOAc	500.0	6.1	517.5	675	0.021	255	0.82	38.4
AcN	499.0	5.6	517.5	715	0.017	235	0.72	41.8
**7g**								
c-hex	504.0	6.1	520.5	630	0.027	355	0.76	27.4
EtOAc	500.0	5.6	518.0	695	0.022	280	0.78	34.9
AcN	499.0	5.3	518.0	735	0.017	245	0.69	40.1
**7h**								
c-hex	504.0	5.9	520.0	610	0.025	320	0.78	30.4
EtOAc	500.0	5.4	517.5	675	0.022	265	0.83	36.9
AcN	499.0	5.0	517.0	695	0.017	230	0.74	42.7

**7i**								
c-hex	504.0	5.5	520.5	630	0.026	335	0.77	29.1
EtOAc	500.0	4.9	517.5	675	0.020	300	0.66	32.6
AcN	499.0	4.5	517.5	715	0.017	245	0.69	40.1
**7j**								
c-hex	504.0	5.8	520.5	630	0.024	310	0.77	31.5
EtOAc	500.0	5.2	517.5	675	0.021	280	0.75	34.9
AcN	499.0	5.0	517.5	715	0.017	230	0.74	36.1

Absorption (λ_{ab}) and fluorescence (λ_{fl}) wavelength; molar absorption at the maximum (ε_{max}); Stokes shift ($\Delta\nu_{St}$); fluorescence quantum yield (ϕ) and lifetime (τ); radiative (k_{fl}) and non-radiative (k_{nr}) rate constants.

c-hex: cyclohexane; EtOAc: ethyl acetate; AcN: acetonitrile

*not soluble in cyclohexane, even in diluted solutions (2 μM)

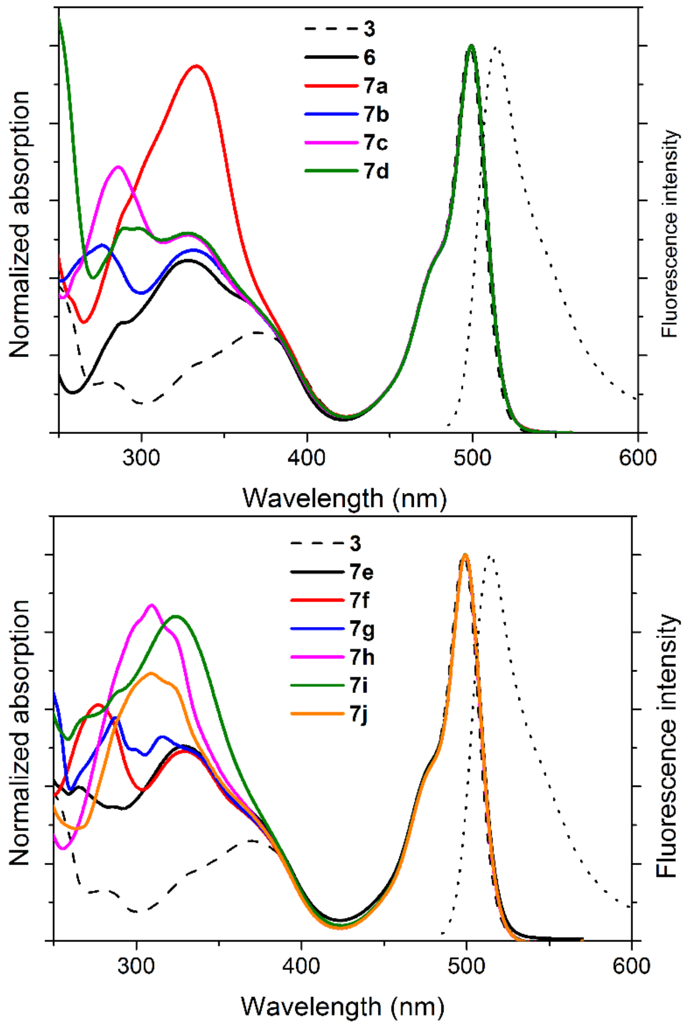

Figure S1. UV-Vis absorption (normalized at the Vis absorption band) spectra of coumarin-BODIPY hybrids in diluted solutions of acetonitrile. The corresponding spectrum of the reference BODIPY **3** (dashed line) is including for comparison. For the sake of simplicity just a representative fluorescence spectra (dotted line) is depicted since the bands position and shape is equal in all the herein tested derivatives regardless of the excitation wavelength (UV or Vis).

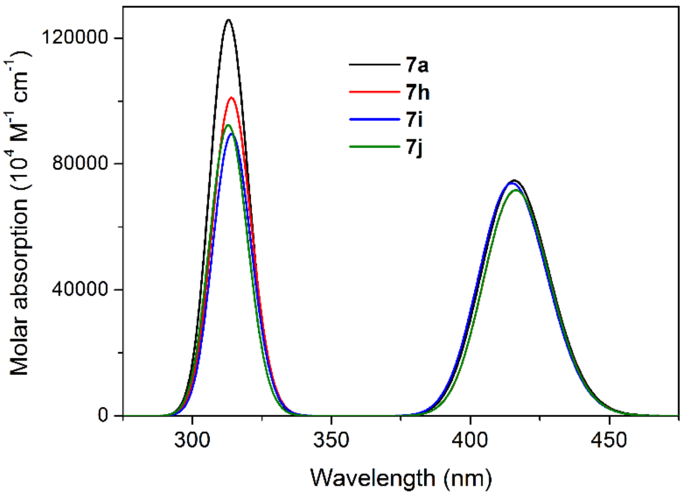

Figure S2. Predicted absorption spectra (td wb97xd/6-311+g*) of coumarin-BODIPY hybrids bearing electron rich moieties at the coumarin fragment.
